# Exploring the Structural and Dynamic Properties of a Chimeric Glycoside Hydrolase Protein in the Presence of Calcium Ions

**DOI:** 10.3390/ijms252211961

**Published:** 2024-11-07

**Authors:** Alberto M. dos Santos, Clauber H. S. da Costa, Manoela Martins, Rosana Goldbeck, Munir S. Skaf

**Affiliations:** 1 Institute of Chemistry and Center for Computing in Engineering and Sciences, University of Campinas (UNICAMP), Campinas 13084-862, SP, Brazil; albertos@unicamp.br (A.M.d.S.); clauberh@unicamp.br (C.H.S.d.C.); 2 School of Food Engineering, University of Campinas (UNICAMP), Campinas 13083-862, SP, Brazil

**Keywords:** glycoside hydrolase, catalytic efficiency, thermostability, enzymatic chimeras

## Abstract

GH10 xylanases and GH62 Arabinofuranosidases play key roles in the breakdown of arabinoxylans and are important tools in various industrial and biotechnological processes, such as renewable biofuel production, the paper industry, and the production of short-chain xylooligosaccharides (XOS) from plant biomass. However, the use of these enzymes in industrial settings is often limited due to their relatively low thermostability and reduced catalytic efficiency. To overcome these limitations, strategies based on enzymatic chimera construction and the use of metal ions and other cofactors have been proposed to produce new recombinant enzymes with improved catalytic activity and thermostability. Here, we examine the conformational dynamics of a GH10-GH62 chimera at different calcium ion concentrations through molecular dynamics simulations. While experimental data have demonstrated improved activity and thermostability in GH10-GH62 chimera, the mechanistic basis for these enhancements remains unclear. We explored the structural details of the binding subsites of Ca^2+^ in the parental enzymes GH62 from *Aspergillus fumigatus* (Afafu62) and a recombinant GH10 from *Cryptococcus flavescens* (Xyn10cf), as well as their chimeric combination, and how negatively charged electron pairing located at the protein surface affects Ca^2+^ capture. The results indicate that Ca^2+^ binding significantly contributes to structural stability and catalytic cavity modulation in the chimera, particularly evident at a concentration of 0.01 M. This effect, not observed in the parental GH10 and GH62 enzymes, highlights how Ca^2+^ enhances stability in the overall chimeric enzyme, while supporting a larger cavity volume in the chimera GH62 subunit. The increased catalytic site volume and reduced structural flexibility in response to Ca^2+^ suggest that calcium binding minimizes non-productive conformational states, which could potentially improve catalytic turnover. The findings presented here may aid in the development of more thermostable and efficient catalytic systems.

## 1. Introduction

Xylanases are enzymes that belong to the glycoside hydrolase (GH) family and play a key role in the hydrolysis of β-1,4 glycosidic bonds in natural polysaccharides, such as arabinoxylans (AXs) [1]. AXs are complex polysaccharides found in cereals like wheat, rice, oats, highland barley, sorghum, and corn and have variations in molecular weight, monosaccharide composition, and structure [1,2,3].

One of the key components of AXs is the presence of arabinose and xylose sugars, which are linked by β-1,4 glycosidic bonds [4]. Endo-β-1,4-xylanases (XLNs) from glycoside hydrolase family 10 (GH10) are responsible for breaking these bonds and releasing the xylose sugars [5]. GH10s have broad substrate specificity, which makes them more effective in hydrolyzing heteroxylans [6]. Additionally, GH10 enzymes have a better ability to cleave glycosidic bonds near the side chains and are less affected by the presence of ramifications [7].

Additionally, α-L-arabinofuranosidases (ABFs) are polysaccharide debranching enzymes that cleave substituents in the α (1→2), α-(1→3), or α-(1→5) bonds in AXs [8]. The hydrolysis of the L-arabinofuranosyl (Araf) residues linked to monosubstituted β-D-Xylp found in AXs can increase the efficiency of plant biomass conversions as a component in enzyme cocktails [2,9,10,11]. These enzymes have the ability to bind to both non-reducing (+2NR, +3NR, +4NR) and reducing (+1 and +2R) substrate extremities, promoting enzymatic cleavage between the −1 and +1 subsites [12,13,14,15].

XLN GH10 and ABF GH62 are essential enzymes for breaking down AXs and play key roles in industrial applications, including biofuel production, animal feed processing, and biotechnological processes involving plant biomass conversion [16,17,18,19]. Moreover, the cocktail combination of both GH62 and GH10 enzymes can be used to achieve more complete hydrolysis of AXs [8,20].

The GH10 family has been found to have thermostable members, with some enzymes having optimum activity at temperatures above 70 °C and even 80 °C [21,22,23]. On the other hand, the GH62 family has not been found to be as thermostable, with some members reaching optimal activity at temperatures not higher than 55 °C [10,24]. In common, the catalytic efficiency of GH10 and GH62 enzymes is often limited by the presence of L-arabinosyl substituents on the AX backbone [25,26]. Therefore, despite their importance, the use of these enzymes in industrial processes is recurrently limited by their low thermostability in industrial settings and modest catalytic efficiency [27,28,29].

The ability to enhance enzyme thermostability and catalytic efficiency has substantial industrial relevance. For example, in biofuel production, more thermostable enzymes reduce the enzyme load required, cutting costs while sustaining yield efficiency [30]. In animal feed processing, enzymes with increased catalytic activity facilitate the breakdown of plant materials, improving nutrient availability and digestion. The use of exogenous enzymes as zootechnical additives has been reviewed, highlighting their role in improving feed digestibility and animal performance [31].

The incorporation of metal ions, such as calcium, has been demonstrated as a promising approach to address those limitations [32,33,34]. It was observed that after incubation at 55 °C for 144 h, in the presence of calcium, GH62 maintained over 80% of its initial activity, a feature that could improve enzyme stability in industrial applications [32]. Furthermore, it was demonstrated that different concentrations of metal ions can alter enzymatic activity, suggesting that some metals can induce changes in protein structure and conformational flexibility, which in turn positively affect the enzyme’s ability to catalyze reactions or resist temperature changes [2,35]. It seems that metal ions can also affect the activity of ABFs, such as AmAraf51 and AmAraf43 enzymes from the anaerobic cellulolytic bacterium *Acetivibrio mesophilus* [36]. This suggests that metal ions may be beneficial for maintaining or increasing enzymatic activity in these GH families.

Another promising approach to enhance thermostability and catalytic efficiency is the construction of chimeric enzymes that combine structural strengths from different enzyme families, potentially overcoming the individual limitations of each parental enzyme [28,37,38]. This approach has been demonstrated to be successful in several studies, where the incorporation of thermostable domains from one enzyme into a less thermostable enzyme has led to an increase in overall thermal stability [39], and the combination of catalytic domains from different enzymes has led to an increase in catalytic efficiency [40,41].

Recently, a study was conducted to design a bifunctional enzyme by fusing an XLN GH10 (Xyn10cf) and an ABF GH62 (Afafu62) to enhance activity and thermostability in the production of xylo-oligosaccharides (XOSs) from AXs, a sustainable source found in agro-industrial wastes [2]. The resulting chimera demonstrated superior specificity and halotolerance for AX. It was also more effective than commercial cocktails, particularly when calcium and manganese ions were added. Also, the thermostability of the ABF module was increased by 10 °C [2,35], enabling a process at higher temperatures and lower enzyme loads, resulting in improved yields of xylobiose and xylotriose by 25% and a maximum yield of 7 g/L XOS from corn hull AX hydrolysis [2].

In this study, we investigated the conformational stability of the Afafu62-Xyn10cf chimera in comparison to its parental components at different calcium ion concentrations using a computational approach. The use of computational methods to predict and design enzymes with improved properties can enhance the efficiency of the chimera construction approach [39,42]. We outline a consistent pattern of conformational changes associated with the interaction of the two distinct subunits and calcium binding, demonstrating structural factors that are critical for the chimera’s mobility. Moreover, we examine the changes in the volume of the substrate-binding site due to the presence of calcium in the chimera and isolated parental enzymes, and correlate these findings with the electrostatic potentials at the solvent-exposed surfaces of the enzymes. These results can help in the development of more thermostable and efficient catalytic chimera systems with improved properties for industrial applications that produce XOS from different sources.

## 2. Results and Discussion

### 2.1. MD Simulation and Ca^2+^–Protein Interactions

It is important to highlight that the wrong start point or protonation state could lead to a different protein structure due to molecular rearrangements with the Ca^2+^ ion. Therefore, the calcium coordination at the catalytic site of the GH62 enzyme was determined by homology modeling using the template 5B6S [12]. The ion is in the channel formed in the center of the GH62 barrel and displays a hepta coordination of five water molecules and His265 (chimera His605), intercalating between Asp29 (chimera Asp369) and Gln86 (chimera Gln426) residues, as described for GH62 enzyme from *Coprinopsis cinerea* (CcAbf62A) [12]. This Ca^2+^ coordination in parental GH62 possibly has a structural function only, although ambiguous results regarding the importance of this ion to other GH62 enzymes were observed [43].

Calcium ions (Ca^2+^) play a crucial role in the stability, thermostability, and catalytic efficiency of glycoside hydrolases. These enzymes are essential components of carbohydrate metabolism and are involved in a variety of biological processes such as plant and animal nutrition, cell wall degradation, and fermentation. The presence of Ca^2+^ ions is known to affect the conformational stability of some enzymes as well as modulate their activity and specificity [9,12].

**Figure 1:**
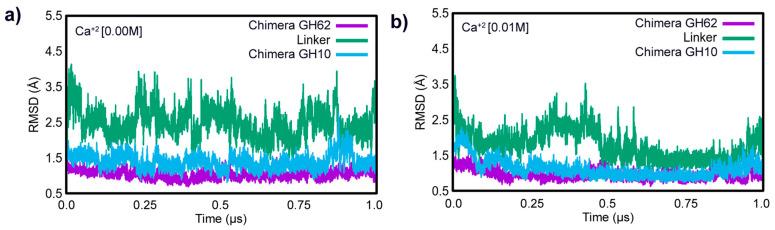
Root mean square deviation (RMSD) of the Afafu62-Xyn10cf chimera subunits over time (**a**) in the absence of calcium ions (Ca^2+^ [0.00 M]) and (**b**) in the presence of 0.01 M calcium ions (Ca^2+^ [0.01 M]). The RMSD of the GH10 subunit is shown in blue, the GH62 subunit in purple, and the linker region in green.

Studies have shown that Ca^2+^ can act as a stabilizing agent by binding to specific sites in GHs, thereby reducing the structural flexibility of the enzymes and increasing their thermal stability [12,43]. In addition, Ca^2+^ ions can also enhance the activity of these enzymes by providing the required ionic strength to promote substrate–enzyme interactions and by affecting the conformation of the active site, thereby facilitating substrate binding [13]. Furthermore, metal ions such as calcium have been recently reported as having a positive effect on the enzymatic activity of GH62 and on the chimera, suggesting that investigating how metal ions can be used to optimize enzymatic activity is an important strategy for more efficient biomass hydrolysis [32,36]. These findings are in line with the observations from our MD simulations, where the presence of Ca^2+^ ions had a significant impact on the conformational dynamics of the chimera and parental proteins studied (Figure 1).

It was observed that the presence of calcium ions has a significant impact on the conformational dynamics of the chimera and parental proteins, as can be seen in the RMSD results obtained over 1.0 µs of MD simulation for each system studied (Table 1 and Figure 1). Increases in Ca^2+^ concentration led to changes in the stability of the chimera protein, specifically in the linker that interconnected the GH10 and GH62 regions. This linker had high mobility and was unstable throughout the MD simulation without ions (Table 1). This was also observed, in general, for the parental protein’s entirety, but at a lower magnitude (Figure 1a). However, at 0.01 M Ca^2+^, the RMSD shifts were below 0.96 Å in the chimera protein and below 1.06 Å in the parental GH62 (Table 1 and Figure 1b), increasing the protein’s stability during the 1.0 µs simulation. When the concentration was increased to 0.1 M, there was a greater variation in the link, but the stability increased significantly after 120 ns (Figure S4). Changes in the overall structure were not observed for the parental GH10 and GH62 enzymes (Figure 1, Table 1, and Figure S4). Results of the RMSD for the MD simulation replicas are available in the Supporting Information (Figures S4 and S5).

Table 1 demonstrates that in both the chimera and the GH62 parental system, the addition of Ca^2+^ at 0.01 M increased the stability of the GH10 and GH62 enzymes. The average RMSD results demonstrate that the structure’s mobility was greater in the absence of Ca^2+^ in the solvent than it was in the presence of 0.01 M Ca^2+^. However, in the presence of an excessive calcium concentration (0.1 M) in the chimeric and parental enzymes, the system showed considerable structural movements. This was probably related to the need for more time for the protein to stabilize in high concentrations of calcium ions.

**Figure 2:**
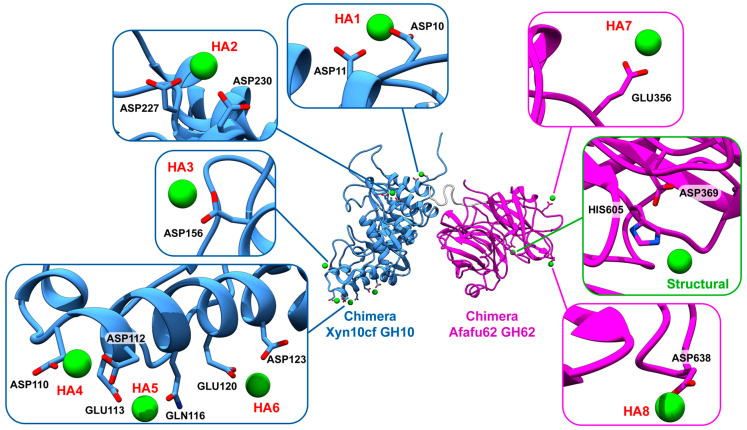
Calcium ion (Ca^2+^) high-affinity (HA) binding regions in the Afafu62-Xyn10cf chimera, illustrating the GH10 (blue) and GH62 (magenta) subunits. The key binding residues are highlighted, showing the specific areas where calcium ions interact with the chimera.

GH10 has negatively charged groups in specific regions that function as binding sites for Ca^2+^ ions; these regions are mainly composed of double residues of Asp, Glu, or some support residues such as Gln and Ser (Figure 2). It is noticeable that Ca^2+^ ions randomly distributed in the solvent bind spontaneously at certain binding regions in the chimera and parental enzymes, even in replicates. Figure 2 shows the calcium binding regions in the chimera and the GH62 catalytic site with structural Ca^2+^ coordination, with key interactions. More information on calcium coordination and additional binding sites can be found in the Supporting Information (Figures S6–S9), which provides a comprehensive view of the Ca^2+^ interactions across both GH10 and GH62. The highest prevalence of ion subsites occurs in six high-affinity (HA) calcium binding regions of GH10 (in blue) and two high-affinity calcium binding sites for GH62, which have favorable regions for binding interactions with Ca^2+^ and keep structural Ca^2+^ in its active site (Figure 2 and Figure S10).

**Figure 3:**
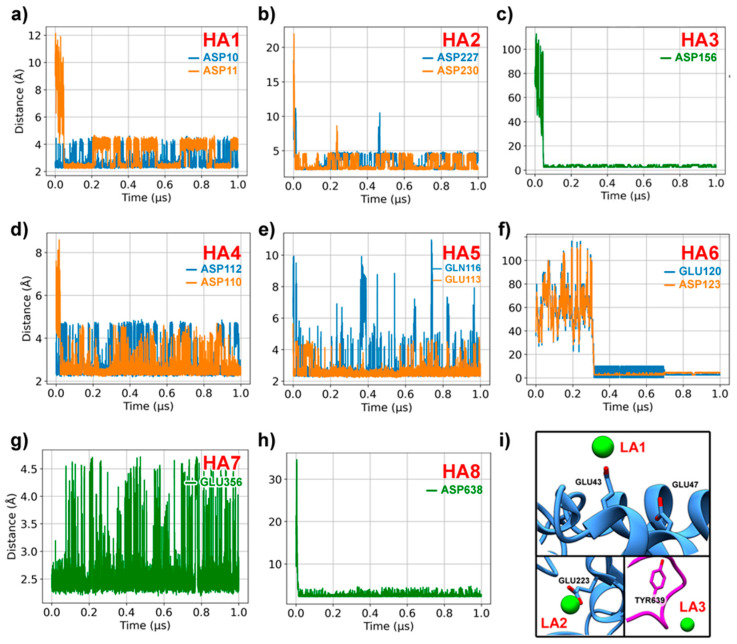
Interaction distance between Ca^2+^ ions and residues on the chimera surface. Distances between Ca^2+^ and (**a**) residues Asp10 (blue) and Asp11 (orange) at the HA1 binding site; (**b**) residues Asp227 (blue) Asp230 (orange) at the HA2 binding site; (**c**) residue Asp156 (green) at the HA3 binding site; (**d**) residues Asp110 (orange) Asp112 (blue) at the HA4 binding site; (**e**) residues Gln116 (blue) Glu113 (orange) at the HA5 binding site; (**f**) residues Glu120 (blue) Asp123 (orange) at the HA6 binding site; (**g**) residue Glu356 (green) at the HA7 binding; site (**h**) residue Asp638 (green) at the HA8 binding site; (**i**) the LA binding sites with GH10 in blue and GH62 in magenta.

As shown in Figure 2, Ca^2+^ ions present in the solvent easily bind to electronegative pairs of Asp and/or Glu residues on the surfaces of the GH subunits. The coordination with His605 kept the structural Ca^2+^ of GH62’s active site stable during the whole MD simulation, mostly with an average distance of 2.80 (0.23) (Figures S8 and S10). It is likely that bonds between these chimera’s surface subsites are responsible for the catalytic improvement in the presence of Ca^2+^. The results demonstrate that once this interaction happened, the Ca^2+^ ion remained bound for most of the MD simulation and, in some circumstances, remained in the subsite until the end of the simulation (Figure 3). Other lower-affinity (LA) binding sites are also distributed through the chimera enzyme surface (Figure 3i). Each panel displays the changes in distance that show how the calcium ion interacted with different residues over the course of the simulation, highlighting how these interactions changed over time and remained stable. The capture of these ions may be explained by the chimera’s unique electrostatic surface, which exposed pairs of Asp and Glu on its surface and interface in the presence of calcium in the solvent.

The MD simulation of the chimera system at a concentration of 0.01 M demonstrated that Ca^2+^ ion binding to the surface is driven by interactions with electronegative residues at the surface, which may favor the stability of the chimera.

**Figure 4:**
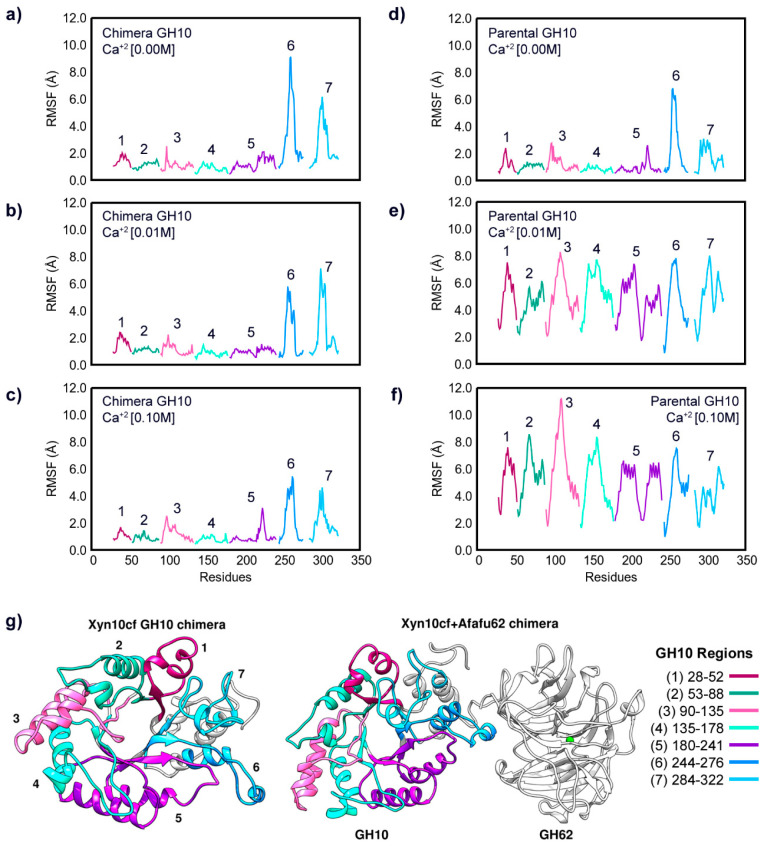
Root mean square fluctuation (RMSF) analysis from MD simulations for the GH10 subunit in different conditions: (**a**) the chimera’s GH10 subunit without calcium ions; (**b**) the chimera’s GH10 subunit at 0.01 M of Ca^2+^; (**c**) the chimera’s GH10 subunit at 0.1 M of Ca^2+^; (**d**) parental GH10 without calcium ions; (**e**) parental GH10 at 0.01 M of Ca^2+^; (**f**) parental GH10 at 0.1 M of Ca^2+^. (**g**) Structural representation highlighting the loop regions of the GH10 subunit in the chimera. The regions are color-coded according to the corresponding RMSF graphs: Region 1 (residues 28–52) in magenta, Region 2 (residues 53–88) in green, Region 3 (residues 90–135) in pink, Region 4 (residues 135–178) in light green, Region 5 (residues 180–241) in purple, Region 6 (residues 244–276) in blue, and Region 7 (residues 284–322) in light blue. RMSF values were derived from the last 200 ns of concatenated MD trajectories.

**Figure 5:**
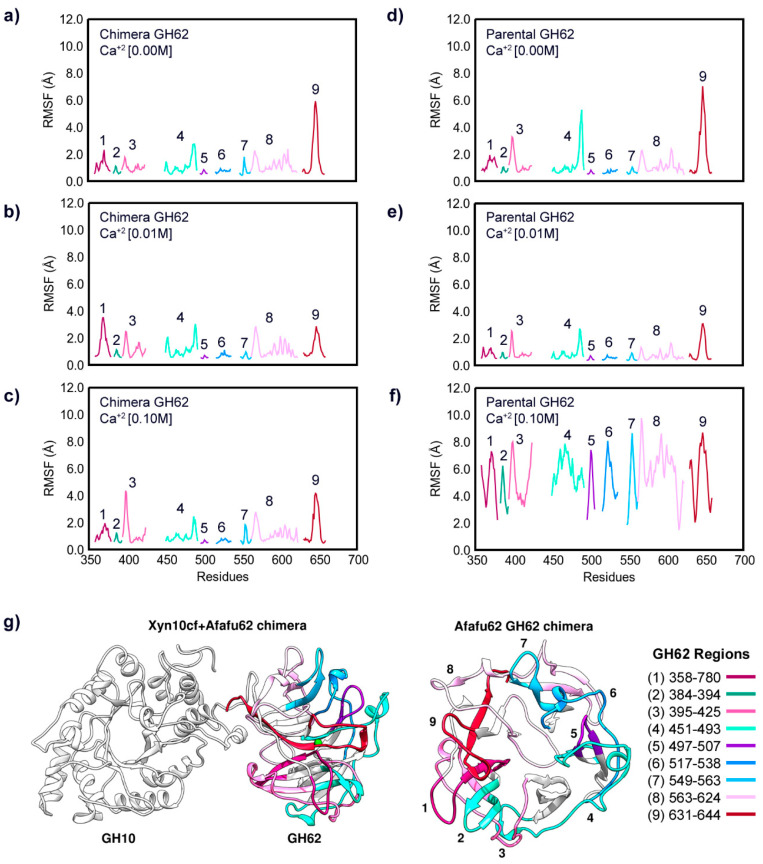
RMSF for the GH62 systems: (**a**) the chimera’s GH62 subunit without calcium ions; (**b**) the chimera’s GH62 subunit at 0.01 M of Ca^2+^; (**c**) the chimera’s GH62 subunit at 0.1 M of Ca^2+^; (**d**) parental GH62 without calcium ions; (**e**) parental GH62 at 0.01 M of Ca^2+^; (**f**) parental GH62 at 0.1 M of Ca^2+^. (**g**) Structural representation highlighting the loop regions of the GH62 subunit in the chimera. The regions are color-coded according to the corresponding RMSF graphs: Region 1 (residues 358–780) in magenta, Region 2 (residues 384–394) in green, Region 3 (residues 395–425) in pink, Region 4 (residues 451–493) in light green, Region 5 (residues 497–507) in purple, Region 6 (residues 517–538) in blue, Region 7 (residues 549–563) in light blue, Region 8 (residues 563–624) in light pink, Region 9 (residues 631–644) in red. RMSF values were derived from the last 200 ns of concatenated MD trajectories. Residues in the parental GH62 have been renumbered to facilitate the visualization and comparison of similar regions across both GH62 systems.

The increase in Ca^2+^ concentration can significantly influence the conformation of glycoside hydrolases (GHs). The Root Mean Square Fluctuation (RMSF) results illustrate how ion binding affects the dynamics of GH10 and GH62. Figure 4 panels (a), (b), and (c) show the RMSF of chimera GH10 at Ca^2+^ concentrations of 0.00 M, 0.01 M, and 0.1 M, respectively, while panels (d), (e), and (f) present the RMSF of the parental GH10 under the same conditions. Similarly, panels (a), (b), and (c) in Figure 5 depict the RMSF of chimera GH62 at Ca^2+^ concentrations of 0.00 M, 0.01 M, and 0.1 M, respectively, while panels (d), (e), and (f) show the parental GH62 in a similar condition.

The RMSF data indicate that the presence of Ca^2+^ ions generally reduce the flexibility of specific loop regions in both GH10 and GH62, as evidenced by the lower RMSF values in Regions 1, 2, 4, and 6 of the enzymes when Ca^2+^ is present. This stabilizing effect is particularly pronounced in the chimera, where higher calcium concentrations enhance stability rather than causing the destabilization observed in the parental enzymes at 0.1 M Ca^2+^ (Figure 4 and Figure 5).

As shown in Figure 4, the presence of Ca^2+^ (0.01 M) reduces the fluctuations of both the chimera and parental GH10 in several regions compared to the absence of Ca^2+^. Notably, the effect of calcium concentration differs between the parental enzymes, with GH62 showing greater sensitivity to Ca^2+^ levels. In the absence of Ca^2+^, parental GH62 exhibits higher fluctuations, and this flexibility is only partially mitigated at 0.01 M Ca^2+^. However, at 0.1 M Ca^2+^, oversaturation of binding sites disrupts structural motifs, particularly in GH10, leading to instability. Notably, Regions 5, 6, and 7 of GH10 exhibit the most pronounced differences between the GH10 chimera and parental forms. This is expected due to their location at the interface of the chimera (panel g). Therefore, while the RMSF data primarily highlight the stabilizing effect of Ca^2+^ ions, they are not sufficient alone to evaluate significant functional differences between these systems beyond the observed stabilization.

In Figure 5, we can see the comparison between the systems regarding the GH62 subunit. Regions 3, 4, 7, 8, and 9 show significant differences in flexibility between the chimera and parental GH62. These regions are critical as they likely play a role in substrate interaction and enzymatic activity. In the absence of Ca^2+^, these regions exhibit higher fluctuations, particularly in parental GH62, suggesting greater inherent flexibility.

In contrast, in the chimera GH62, the RMSF results show smaller fluctuations in the loop regions of the catalytic site compared to the parental GH62. This indicates that the parental enzyme can undergo significant conformational changes affecting access to its active site. Such differences between the chimera and the parental enzyme could enhance substrate access, thereby facilitating catalysis. Specifically, the binding of Ca^2+^ to Asp638 and Asp640 impacts Region 9 of GH62 (Figure 2), which may be crucial for controlling substrate access to the active site. The accessibility of substrates to GH62 is modulated by the behavior of the loops in this region. Moreover, the regions at the chimera’s interface, as shown in panel (e), including Regions 3, 8, and 9, exhibit significant differences in their dynamic behavior in higher concentrations of Ca^2+^. Studies have shown that the Tyr present in loop 9 affects the catalysis, but its role in this process is not yet clear [11]. The parental GH62 exhibits greater flexibility in these interface regions in the absence of Ca^2+^. In the chimera, a significant reduction in flexibility is observed. The parental GH62 exhibits generally higher RMSF values across several regions compared to the chimera GH62 in the absence of Ca^2+^. This reduction in flexibility might enhance substrate accessibility and catalytic turnover but could also imply reduced stability without the stabilizing effect of Ca^2+^.

The comparative RMSF analysis between chimera and parental enzymes at different Ca^2+^ concentrations reveals that the parental enzyme GH62 exhibits greater conformational flexibility in the absence of Ca^2+^. However, the introduction of Ca^2+^ ions leads to a more pronounced stabilization effect in the chimera, particularly in regions crucial for catalytic function. The MD simulation findings align with experimental kinetic data [2], which demonstrate a substantial increase in catalytic efficiency (Kcat/Km) for the GH10-GH62 chimera compared to its parental enzymes. Specifically, the chimera exhibits a catalytic efficiency 1.9 times higher than that of parental GH62 and 8.2 times higher than that of parental GH10. Furthermore, enzyme activity shows a marked increase in the presence of CaCl_2_, particularly at concentrations of 5 mM and 10 mM, reinforcing the functional role of Ca^2+^ in enhancing enzymatic performance [2]. This suggests that the chimeric enzymes are more responsive to the stabilizing influence of Ca^2+^, potentially enhancing their catalytic efficiency by improving substrate access and reducing non-productive conformational states.

### 2.2. Cavity Analysis

**Figure 6:**
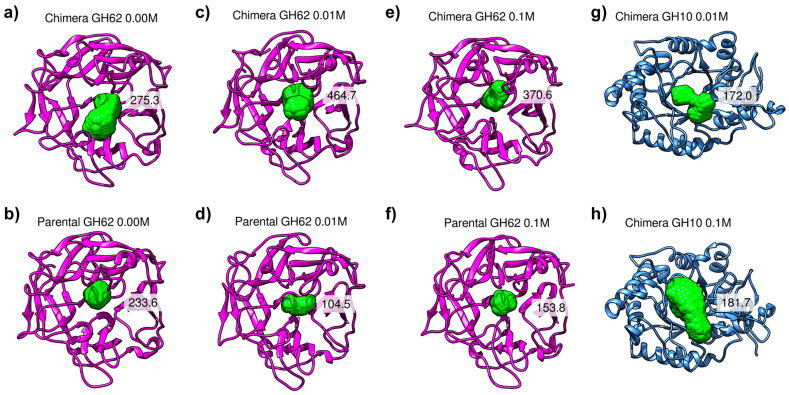
Comparison between parental and chimeric GH62 and GH10 catalytic site volumes under different calcium concentrations: (**a**) the chimera’s GH62 subunit without Ca^2+^; (**b**) parental GH62 without Ca^2+^; (**c**) the chimera’s GH62 subunit at 0.01 M Ca^2+^; (**d**) parental GH62 at 0.01 M Ca^2+^; (**e**) the chimera’s GH62 subunit at 0.1 M Ca^2+^; (**f**) parental GH62 at 0.1 M Ca^2+^; (**g**) the chimera’s GH10 subunit at 0.01 M Ca^2+^; (**h**) parental GH10 at 0.01 M Ca^2+^. The catalytic binding volume is depicted in green. No stable pocket could be identified for the chimera GH10 0.0 M Ca^2+^, chimera GH10 0.1 M Ca^2+^ replica, or any GH10 parental system.

During the analysis of the MD simulations, we observed that the size of the catalytic cavity of the chimera GH62 enzyme was larger in the presence of calcium ions (Figure 6a,c,e and Table 2). Conversely, the parental GH62 exhibited a smaller catalytic cavity in the presence of calcium ions. This finding suggests that one of the chimeric proteins’ contributions is an increased ability to open the catalytic cavity in the presence of calcium ions. These results are in line with those of previous studies on the role of calcium ions in regulating the activity of GHs [44].

Figure 6 and Table 2 provide further insights into the differential impacts of Ca^2+^ concentration on GH10 and GH62 catalytic sites. Notably, while the GH10 chimera displays minimal volume changes with increasing calcium concentrations (0.01 M to 0.10 M), the GH62 systems—both chimera and parental enzymes—demonstrate more pronounced dependence on calcium for catalytic pocket stability and size. Particularly, the chimera GH62 shows a marked increase in cavity volume at 0.01 M Ca^2+^, reaching an average of 464.7 Å^3^, while the parental GH62 exhibits a contrasting reduction to 104.5 Å^3^ under the same conditions. This divergence suggests that the binding of Ca^2+^ ions has a stabilizing effect on the chimera GH62’s catalytic pocket, possibly through interactions at key residues of Region 9 (e.g., Asp638 and Asp640), as described in the RMSF analysis for GH62.

Furthermore, at 0.10 M Ca^2+^, both GH62 variants (chimera and parental) display a reduction in catalytic site volume relative to 0.01 M, although the chimera GH62 retains a significantly larger volume (370.6 Å^3^) compared to the parental GH62 (153.8 Å^3^). This volume retention at higher Ca^2+^ concentrations for the chimera GH62 may indicate structural resilience to ion saturation.

Interestingly, the observed reduction in the catalytic cleft fluctuations or lack of significant differences for the GH10 subunit can be attributed to the inherent stable and solvent-exposed nature of its catalytic site. This openness and stability are evidenced by the absence of a detectable pocket for GH10 in the 0.0 M Ca^2+^ or any GH10 parental system condition, including replicas, throughout the analyzed trajectory (Figures S11 and S12). Unlike GH62, which exhibits an open-to-close conformational flexibility that allows for a defined pocket volume, GH10 maintains a consistently open conformation that is solvent-exposed. This characteristic has been documented in studies highlighting the structural features of GH10 enzymes, where the stable, open conformation is crucial for their catalytic efficiency under varying conditions [28].

Further analysis is needed to fully understand the structural and functional implications of these findings, but it is possible that the increased size of the catalytic site cavity in the presence of calcium ions allows for improved substrate binding and/or increased catalytic efficiency. Additionally, the ability of the GH10 and GH62 enzymes to interact in a manner that results in this alteration in the catalytic site may have important implications for the development of new, more efficient enzymes.

### 2.3. Electrostatic Surface Analysis and Chimera Interface

The interactions between calcium ions and the studied systems were further analyzed by electrostatic potential analysis (Figure S13). The simulation results indicate that the parental GH62 protein is capable of binding two calcium ions. The GH10 protein can make up to 14 simultaneous stable interactions with calcium ions at different sites around the protein (Supplemental Information). The electrostatic potential surface of the two proteins seems to offer the GH62 enzyme new possibilities for binding and interaction with calcium ions, which may directly impact the stability and opening of its binding site.

The increased number of interactions observed in the chimeric protein highlights the potential of protein–protein interactions in modulating the binding of calcium ions and the consequent impact on enzyme activity. Additionally, the changes in the electrostatic potential surface of the GH62 protein could be further explored as a potential mechanism for regulating enzyme activity.

Figure S15 shows the distance from the GH10 and GH62 subunits’ centers of mass in the chimeric systems. Ca^2+^ can affect the stability of the chimera’s protein–protein interfaces, altering its conformational dynamics. A great stability distance between the enzymes was observed for both concentrations of 0.01 M and 0.1 M, whereas the absence of Ca^2+^ may have caused great fluctuations during the MD simulation (Figure S14). Since each GH subunit can move independently, this increase in stability may have a positive impact on substrates’ access to their active sites. Therefore, from a wider perspective, the protein–protein interface ensures the chimera’s overall stability in the presence of calcium ions.

**Figure 7:**
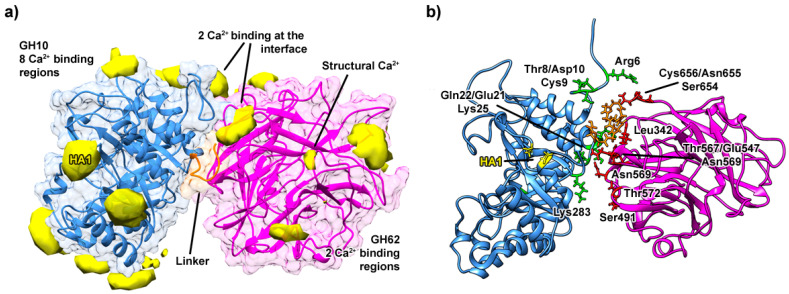
Interface details of the Xyn10cf+Arefu62 chimera at a 0.1 M calcium ion concentration. The GH10 unit is shown in blue, and the GH62 unit is shown in pink. The orange segment indicates the linker region connecting the two units. (**a**) Occupancy analysis of the Ca^2+^ binding regions is depicted in yellow, with more than 50% occupancy based on molecular dynamics (MD) simulation data. (**b**) Main residues involved in the interaction between the GH10 and GH62 subunits are shown in green and red, respectively.

Significant changes in the interface between the GH10 and GH62 subunits can be observed under varying calcium ion concentrations (Figure S15). In the absence of calcium ions (Figure S15a), the interface between GH10 and GH62 is relatively loose, with the catalytic sites perpendicular to each other. Fixed calcium binding sites present in both GH10 and GH62 parental enzymes become inaccessible within the chimera due to the compacted interface formed between the subunits (Figure S15). This structural rearrangement prevents these sites from participating in binding directly, as they are now integrated into the inter-subunit interaction network. When 0.1 M calcium ions were introduced (Figure 7), there was a noticeable shift in the interaction between the subunits, resulting in a more defined interface, with the bottom of the barrel both GH10 and GH62 forming a more stable interface (Figure 7). The same behavior is seen for 0.01 M calcium ion concentration (Figure S15).

Notably, the calcium binding site HA1 located at the interface shows a preference for coordinating with calcium ions rather than forming contacts between GH10 and GH62. In the absence of calcium, this region is actively involved in inter-subunit movement, facilitating dynamic flexibility between units (Figures S6–S9 and S15). However, in the presence of calcium, this region’s ability to interact with other residues is restricted due to calcium coordination, leading to reduced inter-subunit motion and enhanced stability.

The linker region also contributes to the stability of the interface between GH10 and GH62, with its involvement in forming the interface increasing alongside calcium concentration (Figure 7 and Figure S16). At higher calcium levels, the linker plays a greater role in maintaining compactness, supporting the chimera’s overall stability by integrating the subunits into a cohesive structure. This can be seen by the higher number of hydrogen bonds formed from the linker as a bridge between the subunits as the calcium concentration increases (Figure S16).

This interplay between calcium binding, fixed interface regions, and the linker highlights the crucial role of Ca^2+^ ions in modulating the structural dynamics of the chimera and ensuring a stable configuration that optimally supports catalytic function. More details regarding distances between calcium and residues of the chimera enzyme at different calcium concentrations across simulations and replicas are available in Figures S17–S21.

An important question when considering these systems is how to modify the parental enzymes to emulate the enhanced stability and catalytic behavior observed in the chimera. One potential approach is to introduce mutations in the parental enzymes that modulate calcium binding to mimic the binding characteristics seen in the chimera. Our study identified eight high-affinity (HA) and three low-affinity (LA) Ca^2+^ binding sites as promising targets for strategic mutations in both the chimera and parental enzymes.

In GH62, Regions 3 (395–425), 4 (451–493), and 9 (631–644) show higher flexibility, which could be mitigated by mutations that increase calcium affinity or stabilize interactions. For instance, adding calcium-coordinating residues in Regions 3 and 9, or polar substitutions in Region 5, could reinforce structural rigidity and improve substrate positioning, aligning GH62’s behavior more closely with that of the chimera. For GH10, Regions 3 (90–135) and 5 (180–241) exhibit significant fluctuations, especially at higher calcium concentrations. Reducing calcium affinity in these regions—by replacing Asp or Glu with neutral residues—may stabilize the enzyme by limiting non-productive motions and preserving structural integrity, mimicking the chimera’s stability. Mutations that increase Ca^2+^ affinity at certain coordination sites, such as Asp369 and Glu356 in the GH62 subunit, may improve the stability of the catalytic pocket across different Ca^2+^ concentrations. Mutations in regions of the parental enzymes that are located at the chimera interface may enhance its stability at higher calcium concentrations and optimize substrate binding by reducing non-productive conformational changes.

While our study suggests targeted mutations to enhance stability and catalytic efficiency in parental enzymes, further computational and experimental studies are essential to validate and optimize these predictions for practical applications. Overall, by investigating the dynamics of the parental enzymes and identifying mutations that could be introduced into the chimera, it may be possible to achieve improved catalytic activity and stability in the resulting chimeric enzyme.

## 3. Materials and Methods

### 3.1. Molecular Dynamics Simulation

The molecular dynamics (MD) simulations were performed in the Amber20 package (University of California, San Francisco, CA, USA) [45]. The crystal structures of GH10 from *Streptomyces* sp. (PDB code: 3WUG, resolution: 1.9 Å, chain: A) [46] and GH62 from *Coprinopsis cinerea* (PDB code: 5B6S, resolution: 1.7 Å, chain: A) [12] were used as references to construct three molecular systems: (1) Thermostable XLN GH10 from *Papiliotrema flavescens* (Xyn10cf); (2) ABF GH62 from *Aspergillus fumigatus* (Afafu62); (3) the GH10-GH62 (Xyn10cf-Afafu62) chimera linked by a flexible fusion protein sequence (GGGGSGGGGS). The systems were modeled using MODELLER version 10.4 (University of California, San Francisco, CA, USA) [47]. The all-atom force fields were CHARMM36m [48]. Before starting the MD simulations, an assignment of the protonation states of the residues at pH = 5.0 was carried out using the empirical propKa program (Technical University of Denmark, Lyngby, Denmark) [49], based on previously reported optimal conditions for chimera activity [2]. The systems were solvated with an explicit solvation model TIP3P, in a truncated octahedral water-box [50]. A cut-off value of 20.0 Å was used as the distance between the cell wall and the solvated atoms of the system. To neutralize the systems and simulate different calcium concentrations, calcium chloride was also added. Water molecules, hydrogen atoms, ions, and the whole protein structure were minimized through five minimization steps using steepest descent and conjugate gradient with progressive relaxation of restraints [51]. Then, the whole system was heated through 14 heating steps. The 1st heating step was performed at a constant volume for 5 ps (0 to 5K), then from the 2nd step to the 7th step, we used 50 ps to raise the temperature gradually from 5 to 313.5 K (40 °C). Finally, from the 7th heating step, the temperature reached 313.5 K, then remained up until the 14th step. We performed 2 ns of MD simulation to equilibrate the density of the system at constant pressure (1 bar), maintaining the temperature at 313.5 K. The Shake algorithm was applied to all covalent hydrogen bonds in the system [52].

The introduction of a distance limitation between histidine, crystallographic water, and calcium, which was imposed at around 20.0 kcal and gradually decreased during the equilibration stage, allowed for the maintenance of the calcium coordination of the GH62 active site during the equilibration stage. No constraints were used during the production stage of any of the MD simulations.

We performed 1.0 µs of MD simulation using the NPT ensemble for each of the systems for three different calcium chloride concentrations: 0.0 M, 0.01 M, and 0.1 M. The 0.01 M concentration was selected based on previous experimental studies that demonstrated improved enzymatic efficiency in the chimera at this level, particularly improving catalytic activity compared to that of parental enzymes [2]. The 0.1 M concentration, albeit exceeding physiological levels, was included to explore additional potential calcium binding sites on the enzyme’s surface. For each of the nine systems (chimera, GH62, and GH10 at three calcium concentrations), we performed a replicate simulation with a different starting configuration, resulting in a total of 18 MD simulations of 1.0 µs. We conducted a principal component analysis (PCA) to assess structural similarities across simulations and their replicates as described previously [53]. This analysis confirmed that the minimum representative structures obtained from each MD simulation and its replicate were highly consistent (Figures S1–S3). Finally, the root mean square deviation (RMSD) and root mean square fluctuation (RMSF) values of chimera and parental structures were obtained from the heavy atom’s backbone, as were the interactions between the subunits of the chimera enzyme. The RMSF values were obtained using concatenated trajectories from the final 200 ns of each MD simulation and its respective replicate.

### 3.2. Pocket Volume Analysis

The analysis of the volume variation of the catalytic pocket during all the MD simulations was tested with MDpocket 4.2 [54]. Fpocket was used to perform initial pocket detection, and its output was a useful reference with which to prepare the input pocket file needed for the analysis performed by MDpocket on the trajectories. The first step of the analysis consisted of the definition of a bounding box surrounding a specific region of the structure, which was inspected to identify pockets on the protein surface of the reference structure. The identified pockets were tracked along the trajectory by MDpocket, which calculated their volume and other geometric properties (like the solvent accessible surface area or an index of hydrophobicity). These methods can be used entirely unsupervised over the entire surface, but depending on the protein size, the computational slowdown can be significant; additionally, we were only interested in evaluating the plasticity of the active site. The starting region was chosen by selecting a list of residues that covered the catalytic site of each target independently. The trajectory had to be in line with the original reference structure for MDpocket to be able to track a particular pocket throughout the dynamics. Alignment was performed using the carbon atoms of the whole protein’s residues. To improve statistical reliability, we extended the pocket and distance analyses to the full 1.0 µs time interval.

## 4. Conclusions

In this work, we performed MD simulations of the catalytic core domains of GH10 and GH62 enzymes in their native forms and of a GH10-GH62 chimera recently constructed using recombinant techniques [2]. The simulation results reported here provide a more detailed view of the molecular basis for the experimentally observed increase in the stability and catalytic activity of the chimera compared to those of the individual parental enzymes in the presence of calcium ions. We compared the overall structure stability and conformational flexibility of the parental and chimeric enzymes under different calcium concentrations, examined the volumes of the enzyme’s catalytic site, and investigated the electrostatic surface of the proteins. The present study found that the presence of calcium ions (Ca^2+^) has a significant impact on the conformational dynamics of the GH10-GH62 chimera and parental proteins. The RMSD results obtained from molecular dynamics simulations showed that increases in Ca^2+^ concentration led to changes in the stability of the chimera protein, specifically in the linker that interconnects the GH10 and GH62 regions, supporting improved protein stability. Additionally, we identified negatively charged groups in specific GH10 regions that act as calcium binding sites, which likely contribute to this stabilization. Our study also observed that increased Ca^2+^ concentration can modulate the substrate’s ability to access GH62’s active site by affecting loop flexibility. It is important to note that these findings are specific to the GH10-GH62 chimera and its parental enzymes. While our results provide insights into calcium’s role in stabilizing and modulating this particular system, broader applications may require further validation. Nevertheless, this study highlights the potential of calcium binding in enzyme stabilization and may have important implications for the development of new, more stable, and more catalytically efficient enzymes.

## Figures and Tables

**Table 1:** RMSD results after 1.0 µs of MD simulation for chimera system subunits and parental GH10 and GH62 enzymes. Standard deviations are shown in parentheses. Values are given in Å.

[Ca^2+^]	Chimera GH10	Chimera Linker	Chimera GH62	Parental GH10	Parental GH62
0.00 M	1.39 (0.21)	2.49 (0.41)	1.03 (0.12)	1.04 (0.23)	1.23 (0.18)
0.01 M	1.12 (0.26)	1.79 (0.42)	0.96 (0.14)	1.02 (0.21)	1.06 (0.14)
0.10 M	1.25 (0.17)	1.56 (0.82)	1.07 (0.25)	1.22 (0.21)	1.19 (0.15)

**Table 2:** Cavity analyses obtained from the 2 μs of concatenated MD trajectories. Standard deviations are shown in parentheses. Values are given in Å^3^. Values are averaged across both simulation and replica. No stable pocket could be identified for GH10 parental systems.

[Ca^2+^]	Chimera GH10	Chimera GH62	Parental GH62
0.00 M	-	275.3 (106.5)	233.6 (55.9)
0.01 M	172.0 (112.1)	464.7 (109.0)	104.5 (52.2)
0.10 M	181.7 (134.3)	370.6 (127.1)	153.8 (48.2)

## Data Availability

Data is contained within the article and Supplementary Material.

## Supplementary Materials

The following materials are available online at https://www.mdpi.com/article/10.3390/ijms252211961/s1.
