# Supplementary material for: Exploring the Structural and Dynamic Properties of a Chimeric Glycoside Hydrolase Protein in the Presence of Calcium Ions
Source: Int J Mol Sci. 2024 Nov 7;25(22):11961. doi: 10.3390/ijms252211961 (PMC11594105; doi:10.3390/ijms252211961)
Supplement: Supplementary file 1 [file ijms-25-11961-s001.zip › ijms-3245267-supplementary.pdf]

Exploring the Structural and Dynamic Properties of a Chimeric Glucosyl Hydrolase Protein in the Presence of Calcium Ions

Alberto M. dos Santos[a], Clauber H. Costa[a], Manoela Martins[b], Rosana Goldbeck[b], Munir S. Skaf[a]*

[a] Institute of Chemistry and Center for Computer in Engineering and Sciences, University of Campinas (UNICAMP), Campinas 13084-862, São Paulo, Brazil

[b] School of Food Engineering, University of Campinas, Campinas, Brazil

*correspondence should be addressed:

Munir S. Skaf: skaf@unicamp.br

Representative PDB structures

We made publicly available the representative PDB structures and other simulation data on GitHub (https://github.com/albertomds/GHchimera).

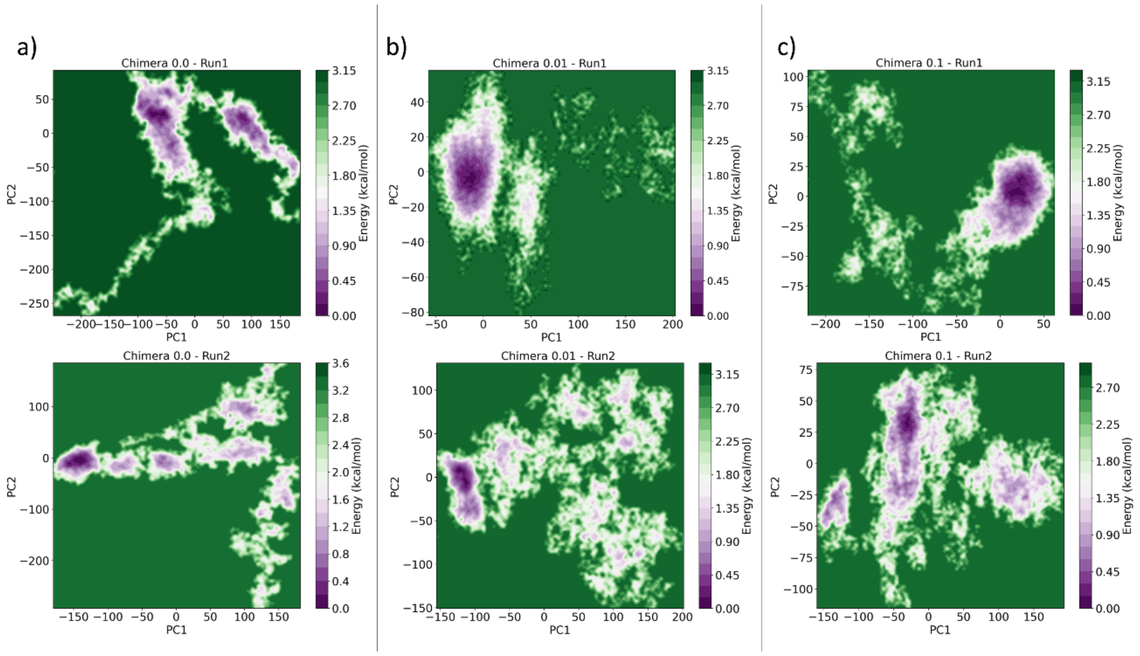

Figure S1. Principal Component Analysis (PCA) with Free Energy Landscape (FEL) representation for the Chimera Afafu62-Xyn10cf, analyzed using alpha-carbon atoms at three different concentrations: a) Chimera 0.0, b) Chimera 0.01, and c) Chimera 0.1, each assessed in two molecular dynamics simulations (Run1 and Run2). Each plot illustrates the distribution along the primary conformational axes (PC1 and PC2), with color gradients reflecting the relative free energy (kcal/mol) within the FEL. Purple regions correspond to lower-energy conformations, indicating stable states, while green regions represent higher-energy, less stable conformations. Structures are available in GitHub.

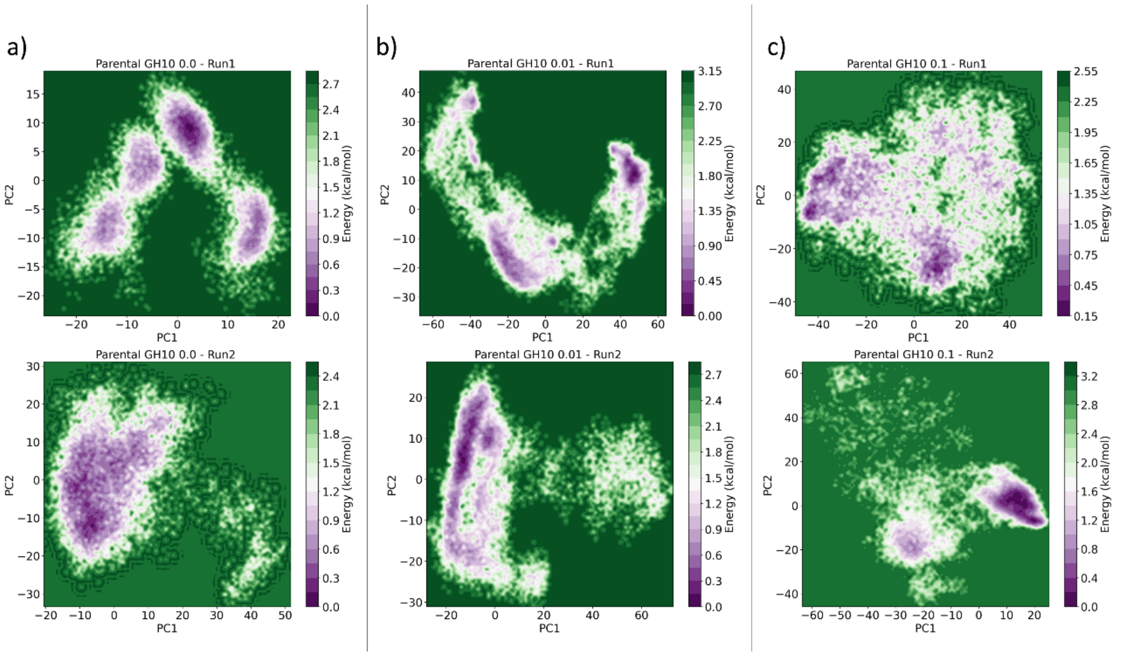

Figure S2. Principal Component Analysis (PCA) with Free Energy Landscape (FEL) representation for the Xyn10cf GH10, analyzed using alpha-carbon atoms at three different concentrations: a) Chimera 0.0, b) Chimera 0.01, and c) Chimera 0.1, each assessed in two molecular dynamics simulations (Run1 and Run2). Each plot illustrates the distribution along the primary conformational axes (PC1 and PC2), with color gradients reflecting the relative free energy (kcal/mol) within the FEL. Purple regions correspond to lower-energy conformations, indicating stable states, while green regions represent higher-energy, less stable conformations. Structures are available in GitHub.

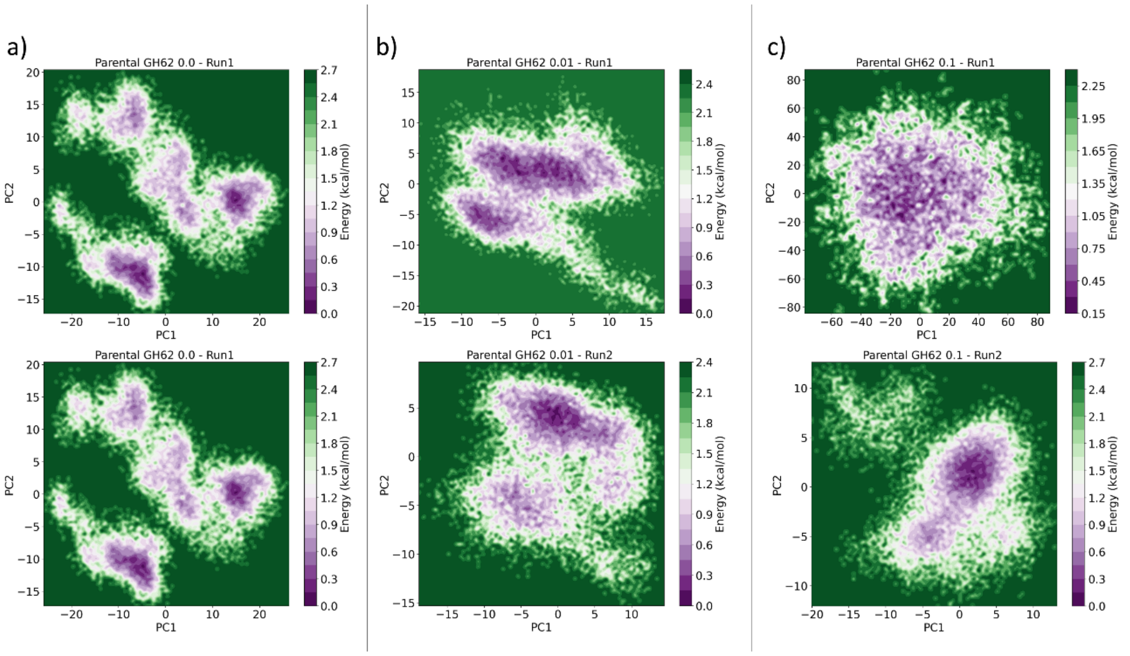

Figure S3. Principal Component Analysis (PCA) with Free Energy Landscape (FEL) representation for the Afafu62 GH62, analyzed using alpha-carbon atoms at three different concentrations: a) Chimera 0.0, b) Chimera 0.01, and c) Chimera 0.1, each assessed in two molecular dynamics simulations (Run1 and Run2). Each plot illustrates the distribution along the primary conformational axes (PC1 and PC2), with color gradients reflecting the relative free energy (kcal/mol) within the FEL. Purple regions correspond to lower-energy conformations, indicating stable states, while green regions represent higher-energy, less stable conformations. Structures are available in GitHub.

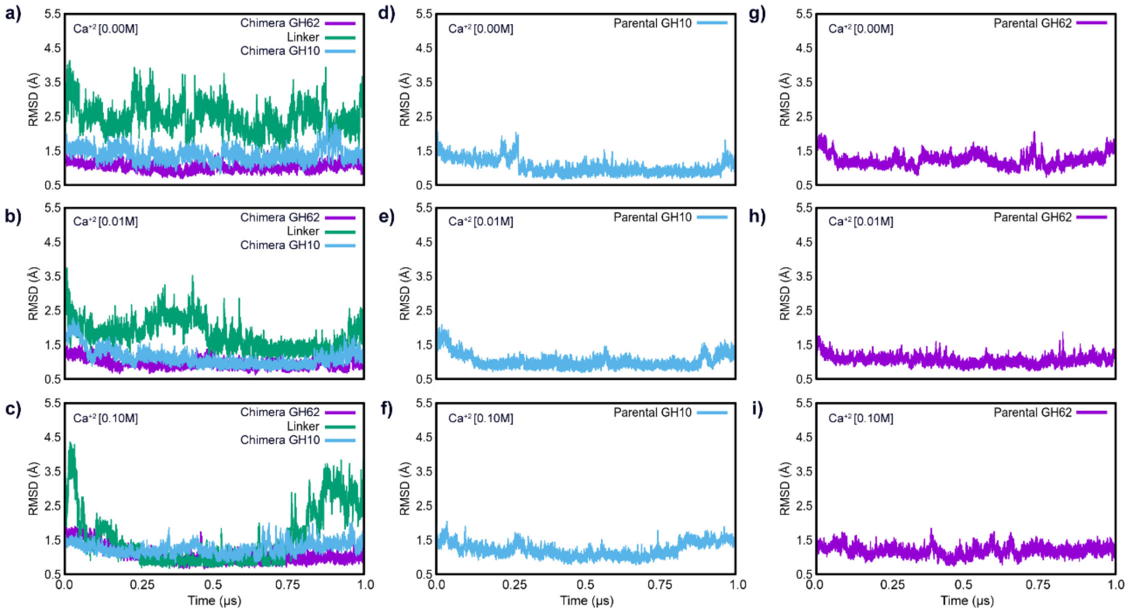

Figure S4. Root mean square deviation (RMSD) for the Afafu62-Xyn10cf chimera and parental enzymes in (a) Chimera without calcium, (b) Chimera at 0.01M of Ca^{+2}, (c) Chimera at 0.1M of Ca^{+2}, (d) Parental GH10 without calcium, (e) Parental GH10 at 0.01M of Ca^{+2}, (f) Parental GH10 at 0.1M of Ca^{+2}, (g) Parental GH62 without Ca^{+2}, (h) Parental GH62 at 0.01M of Ca^{+2}, and (i) Parental GH62 at 0.1M of Ca^{+2}.The GH10, GH62, and Linker subunits are depicted in blue, purple, and green lines, respectively.

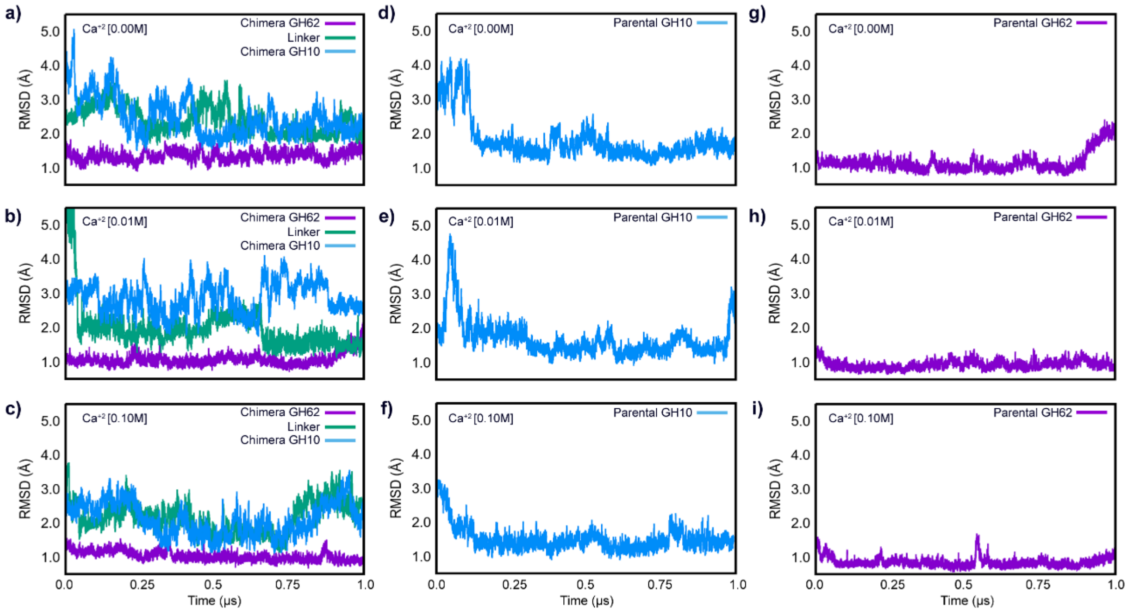

Figure S5. Root mean square deviation (RMSD) for the replicas of Afafu62-Xyn10cf chimera and parental enzymes in (a) Chimera without calcium, (b) Chimera at 0.01M of

Ca^{+2}, (c) Chimera at 0.1M of Ca^{+2}, (d) Parental GH10 without calcium, (e) Parental GH10 at 0.01M of Ca^{+2}, (f) Parental GH10 at 0.1M of Ca^{+2}, (g) Parental GH62 without Ca^{+2}, (h) Parental GH62 at 0.01M of Ca^{+2}, and (i) Parental GH62 at 0.1M of Ca^{+2}.The GH10, GH62, and Linker subunits are depicted in blue, purple, and green lines, respectively.

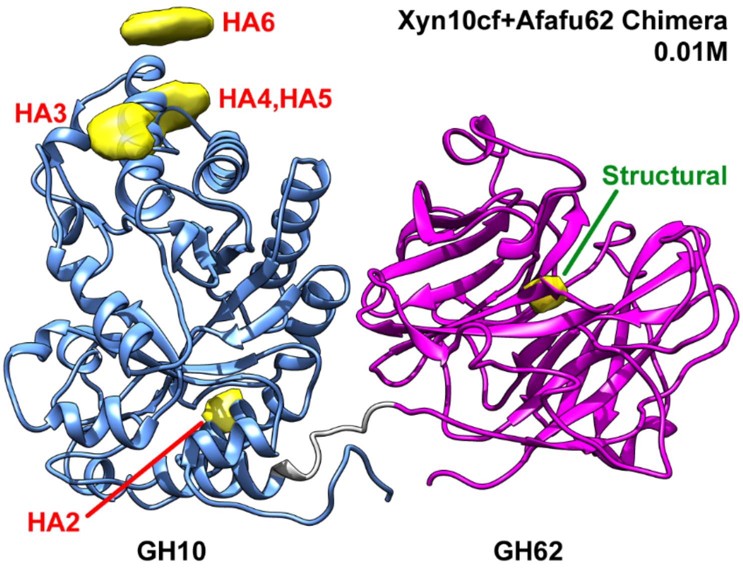

Figure S6. Structural representation of the Xyn10cf+Arefu62 chimera at 0.01 M calcium ion concentration. The GH10 unit is shown in blue, and the GH62 unit is shown in pink. The white segment indicates the linker region connecting the two units. Ca^{+2} binding regions are depicted in yellow, with more than 50% occupancy based on molecular dynamics (MD) simulation data.

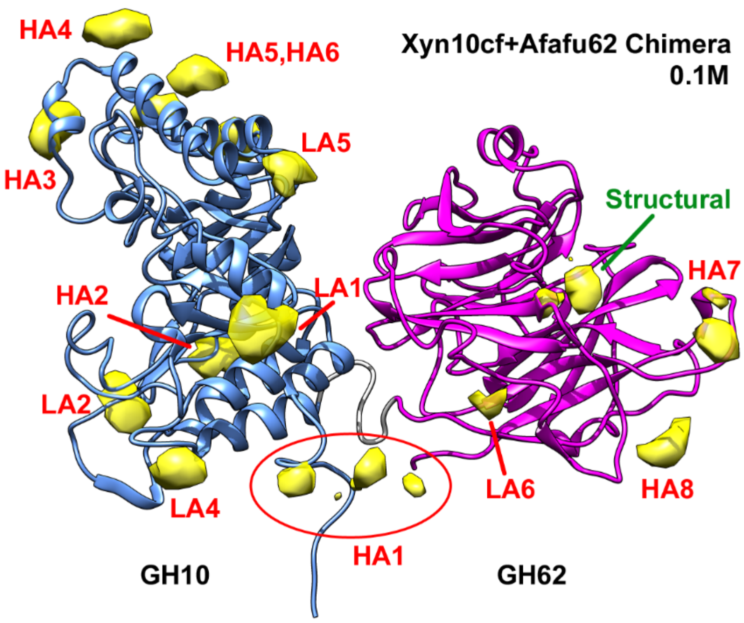

Figure S7. Structural representation of the Xyn10cf+Arefu62 chimera at 0.1 M calcium ion concentration. The GH10 unit is shown in blue, and the GH62 unit is shown in pink. The white segment indicates the linker region connecting the two units. Ca^{+2} binding regions are depicted in yellow, with more than 50% occupancy based on molecular dynamics (MD) simulation data.

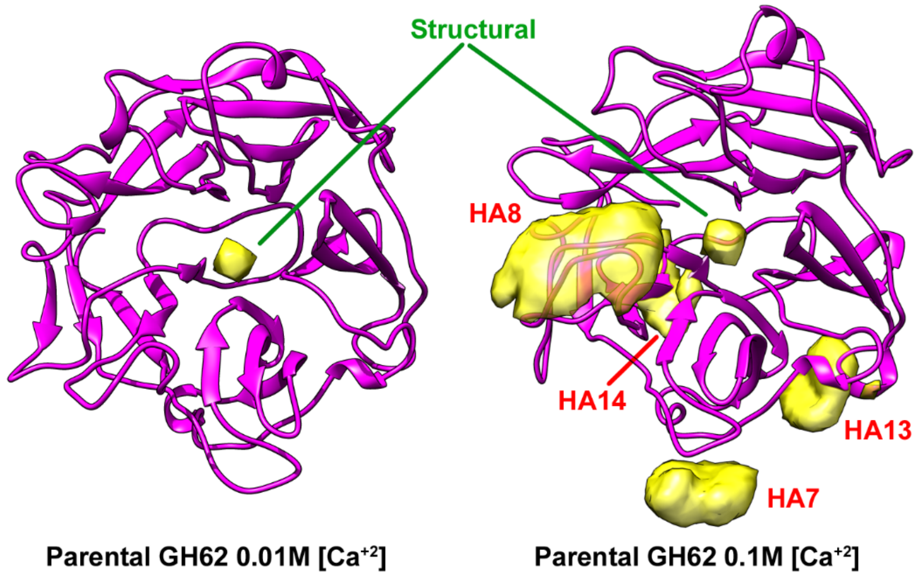

Figure S8. Structural representation of the parental GH62 at 0.01 M (left) and 0.1 M (right) calcium ion concentrations Ca^{+2} binding regions are depicted in yellow, with more than 50% occupancy based on molecular dynamics (MD) simulation data.

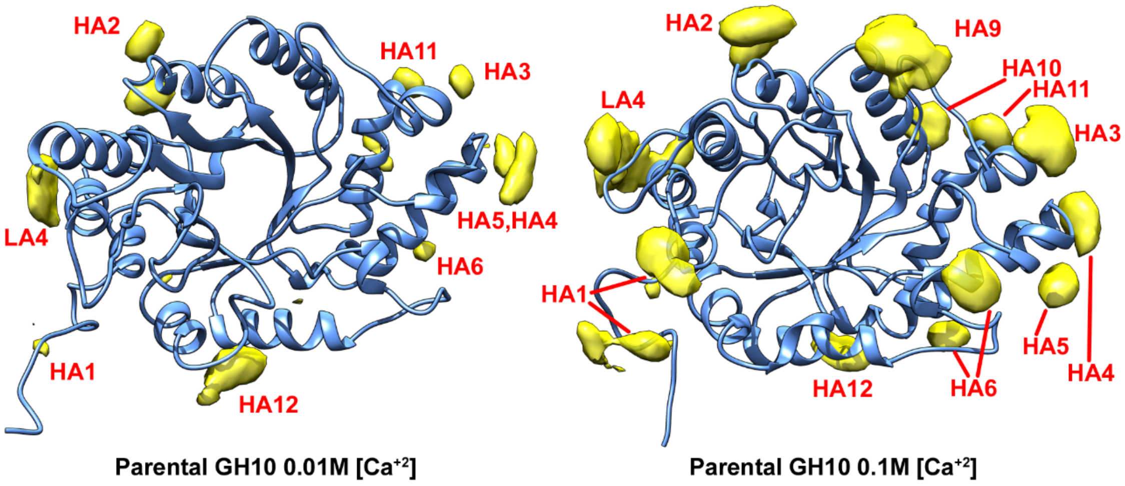

Figure S9. Structural representation of the parental GH10 at 0.01 M (left) and 0.1 M (right) calcium ion concentrations Ca^{+2} binding regions are depicted in yellow, with more than 50% occupancy based on molecular dynamics (MD) simulation data.

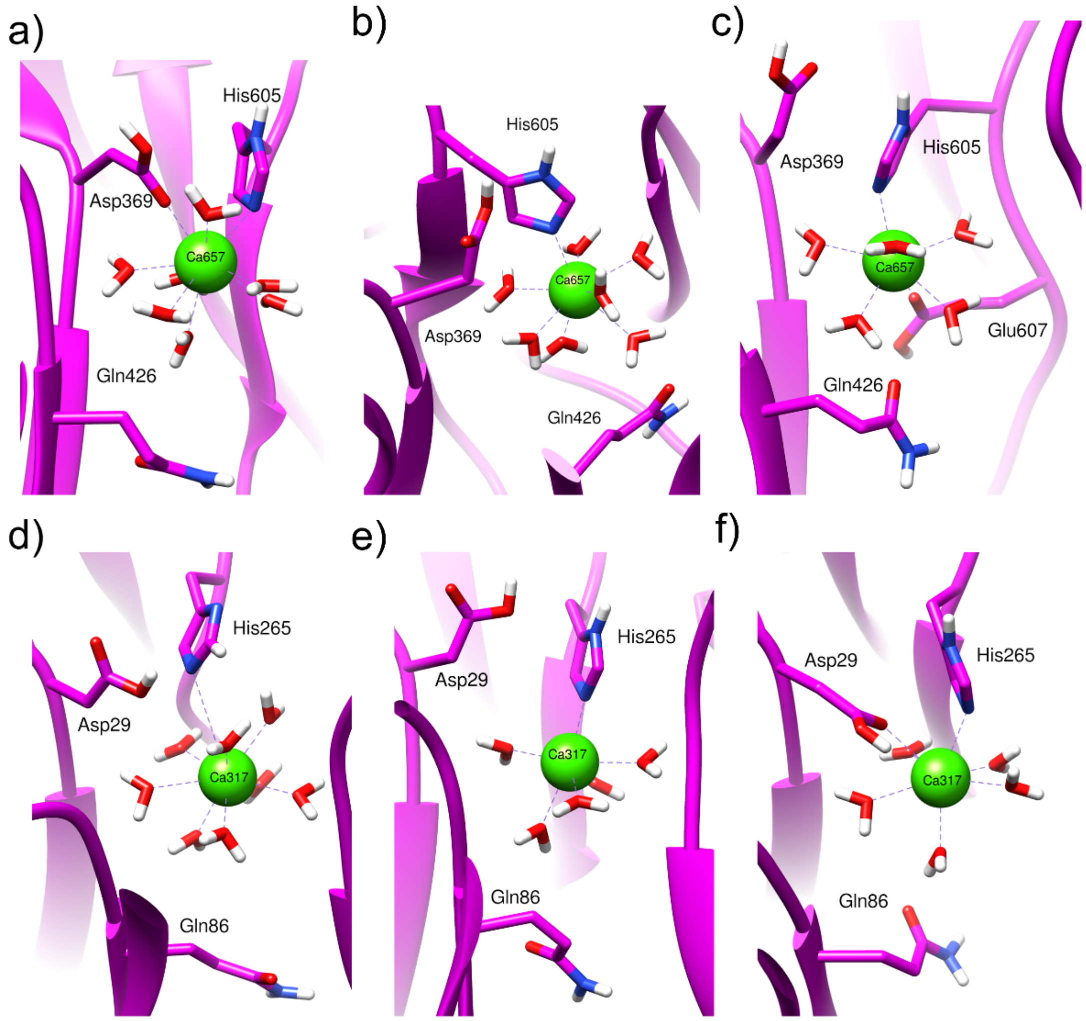

Figure S10. Coordination of the structural calcium ion (Ca^{2}□, in green) in different systems of the Chimera Afafu62-Xyn10cf and the parental GH10 and GH62 at various calcium concentrations: a) Chimera 0.00M; b) Chimera 0.01M; c) Chimera 0.1M; d) Parental GH62 0.00M; e) Parental GH62 0.01M; and f) Parental GH62 0.1M.

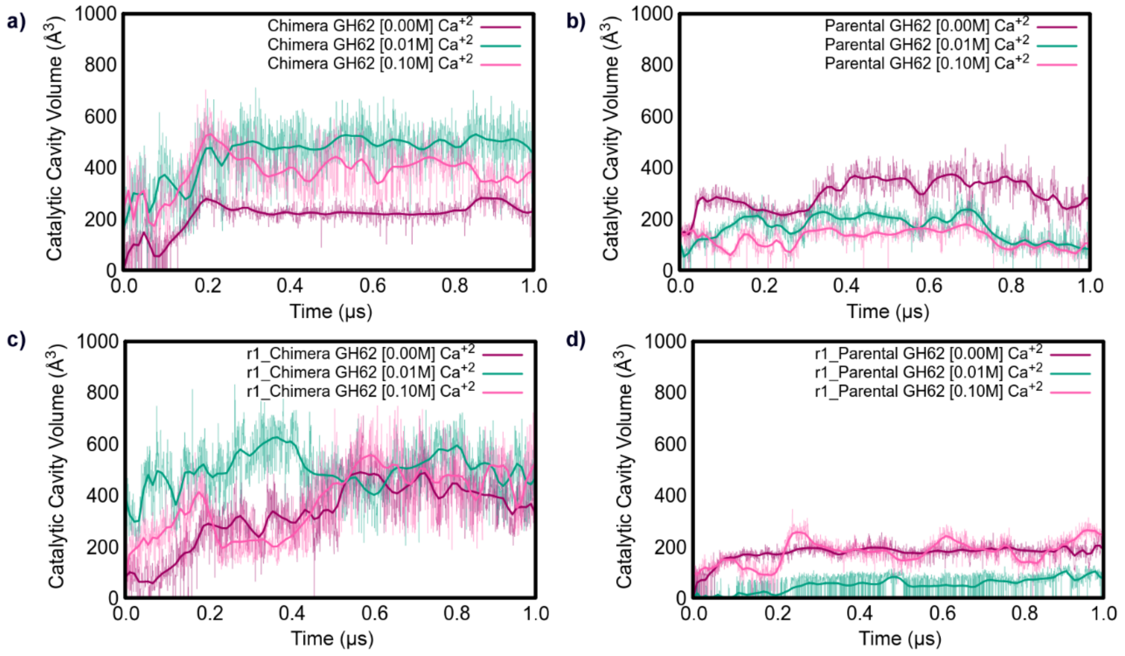

Figure S11. Comparison between Chimera and Parental GH62 enzymes' volume of the catalytic sites. (a) Chimera's GH62 subunit (b) Parental GH62 (c) Chimera's GH62 replica (d) Parental GH62 replica. Cavity analyses were obtained over the entire 1 μs MD simulation.

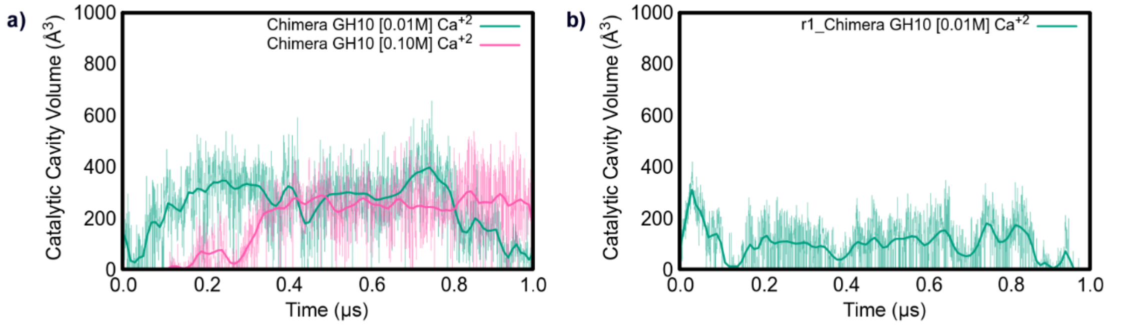

Figure S12. Comparison between Chimera and Parental GH10 volume of the catalytic sites. (a) Chimera GH10 subunit (b) Chimera GH10 replica. Cavity analyses were obtained for the 1μs of MD simulation. No stable pocket could be identified using MDpocket for the chimera 0.0M Ca^{2}□, chimera 0.1M Ca^{2}□ replica, or any parental system condition.

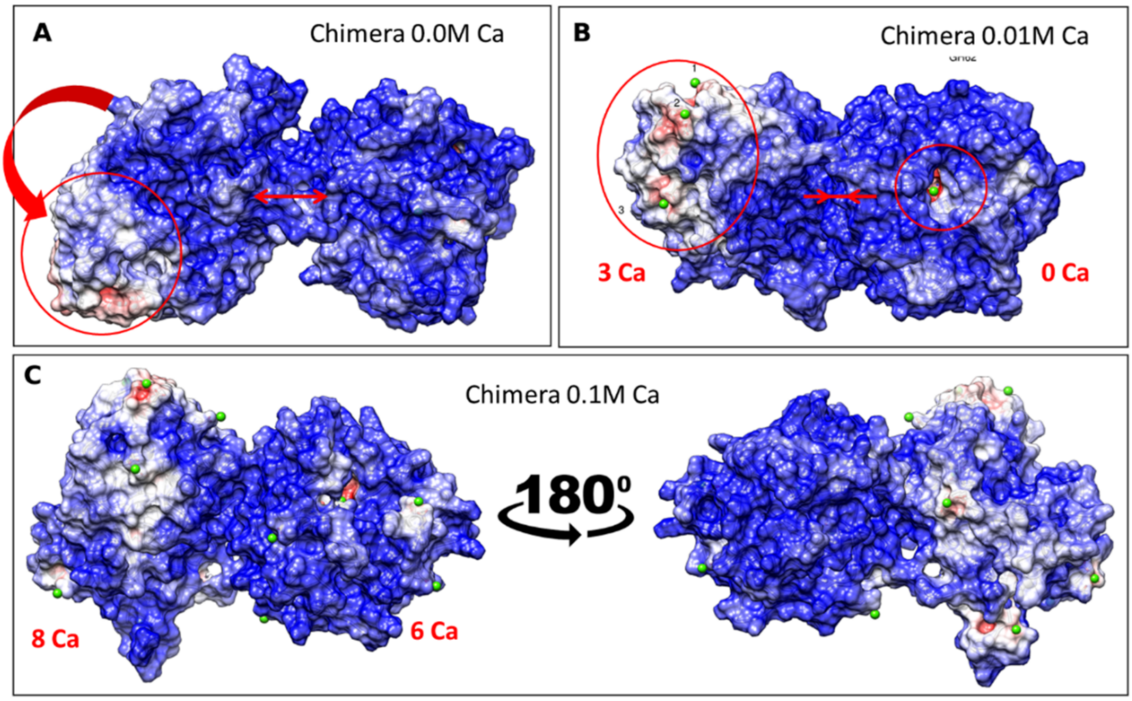

Figure S13. Electrostatic surface potential changes in the Afafu62-Xyn10cf chimera at varying calcium ion concentrations, calculated using the Adaptive Poisson-Boltzmann Solver (APBS). Blue represents positive charge, red indicates negative charge, and white denotes neutral charge on the surface. (a) Chimera without calcium in the solvent (b) Chimera at 0.01M Ca2+, highlighting the electrostatic changes and calcium binding sites. (c) Chimera at 0.1M Ca2+, with a 180-degree rotated view to display the electrostatic surface and calcium binding sites on both sides of the protein. Red circles and arrows indicate significant regions of electrostatic potential variation and calcium binding locations. The number of bound calcium ions is noted in red for each condition.

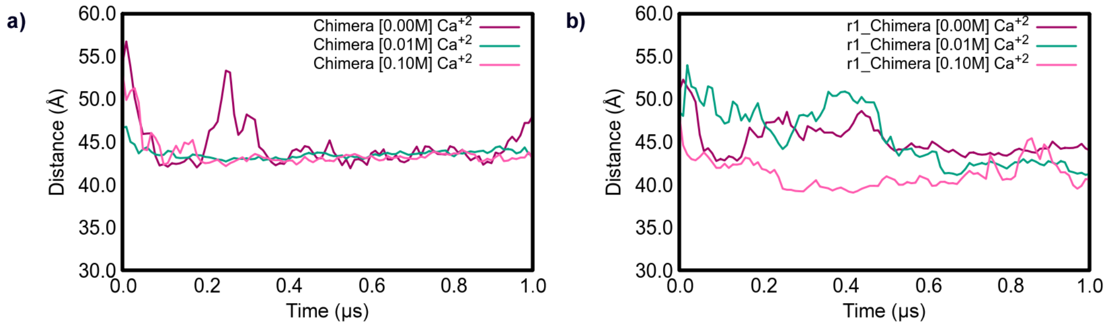

Figure S14. The distance between the subunit's centers of mass in the chimeric system at different calcium ion concentrations on the a) 1 μs of MD simulation b) 1 μs of MD simulation replica.

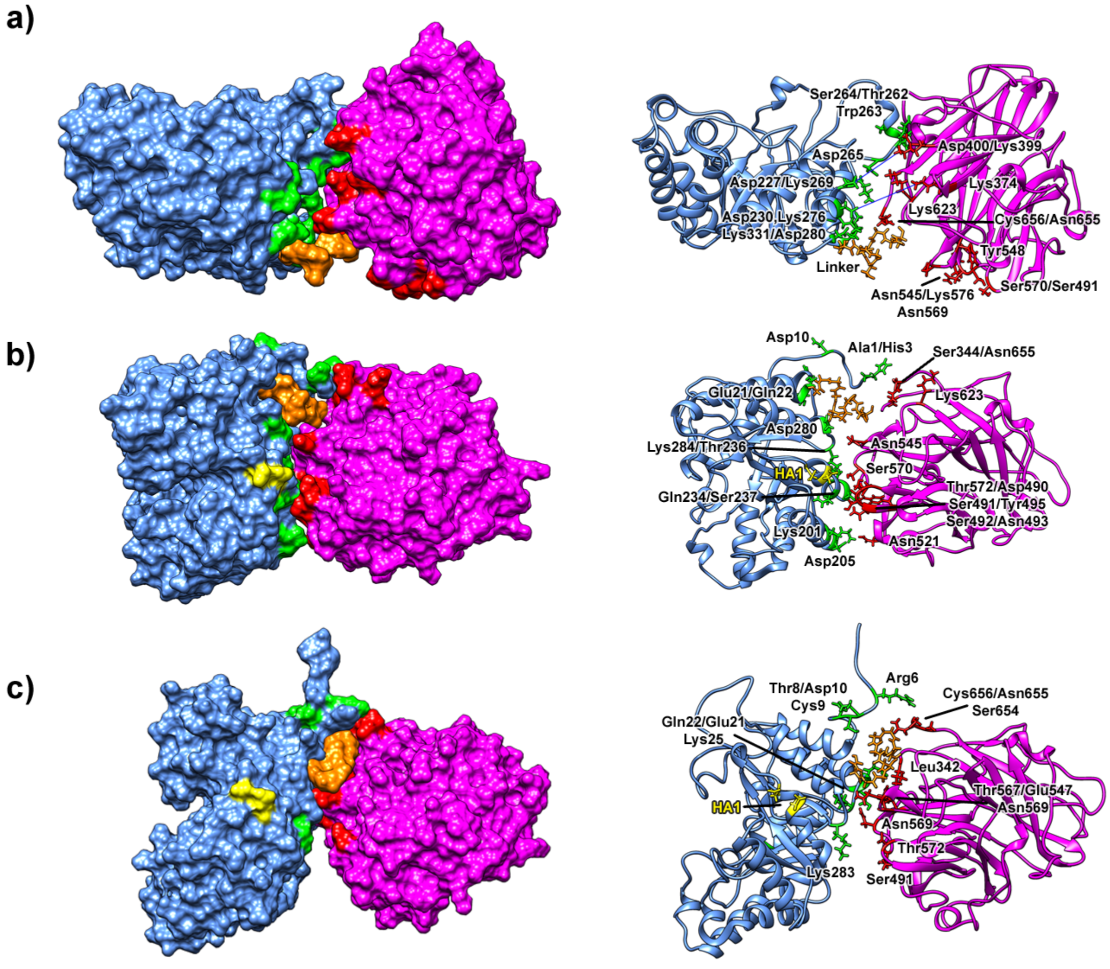

Figure S15. Comparison between parental and chimeric GH62 and GH10 interfaces obtained from representative structures of a) Chimera 0.0M b) Chimera 0.01M c) Chimera 0.1M. The GH10 unit is shown in blue, and the GH62 unit is shown in pink.

The orange segment indicates the linker region connecting the two units. The major Ca+2 binding region is depicted in yellow.

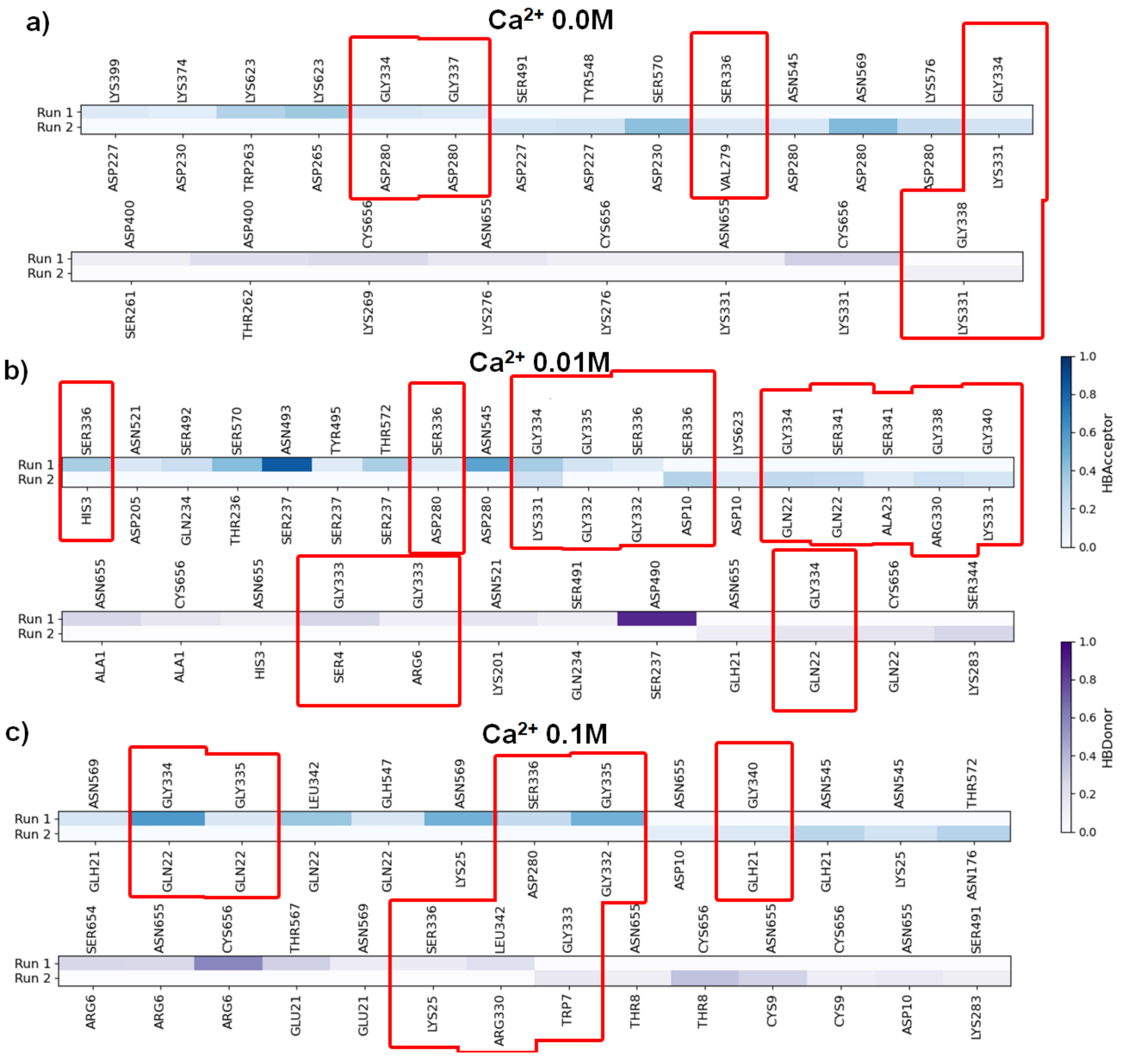

Figure S16. Protein-Protein Interaction Fingerprint for the Chimera interface, GH10 (lower residues in the graph), and GH62 (upper residues in the graph) at different calcium concentrations: (a) Chimera 0.00M; (b) Chimera 0.01M; (c) Chimera 0.1M. The color variations reflect the frequency of interactions over a period of 1 µs for each system studied. The types of interactions are: Hydrogen Bond Acceptor (HBAcceptor) interactions in shades of blue, and Hydrogen Bond Donor (HBDonor) in shades of purple, interactions. Interactions with Linker residues are highlighted in red.

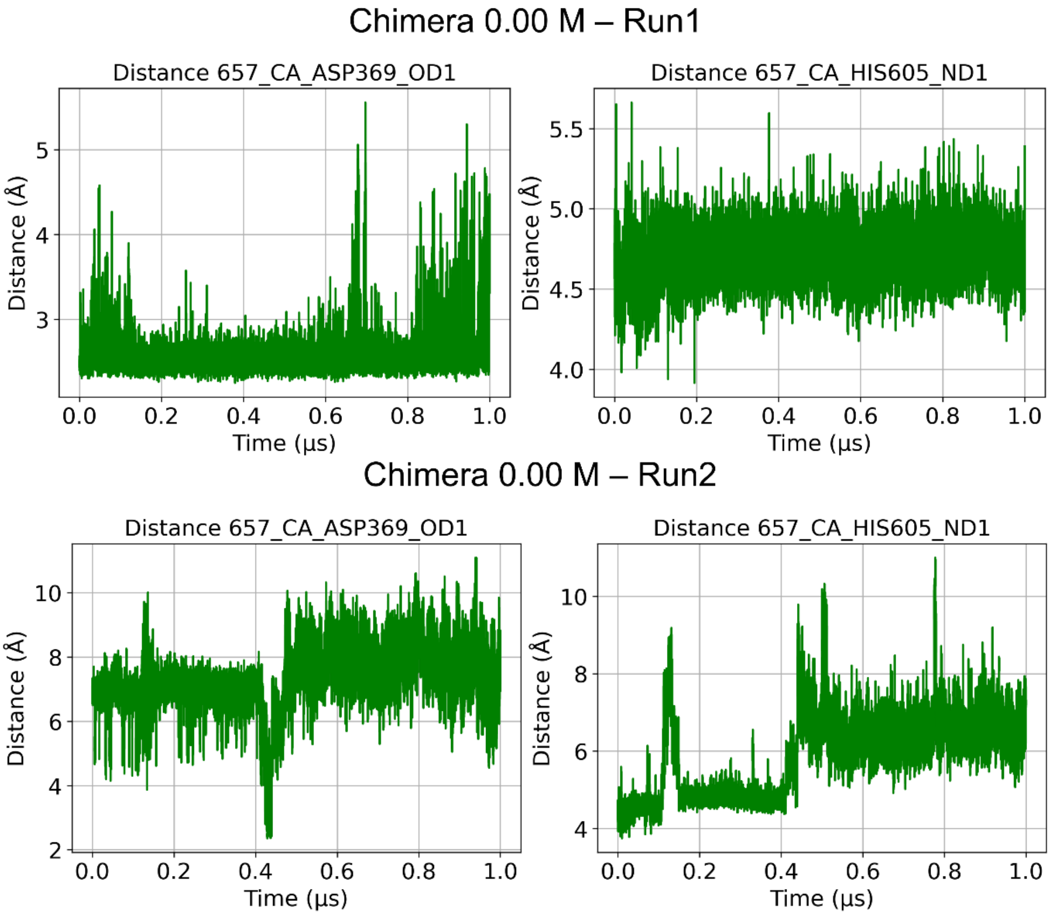

Figure S17. Distance between the structural calcium and the residues of the GH62 chimera without calcium ion.

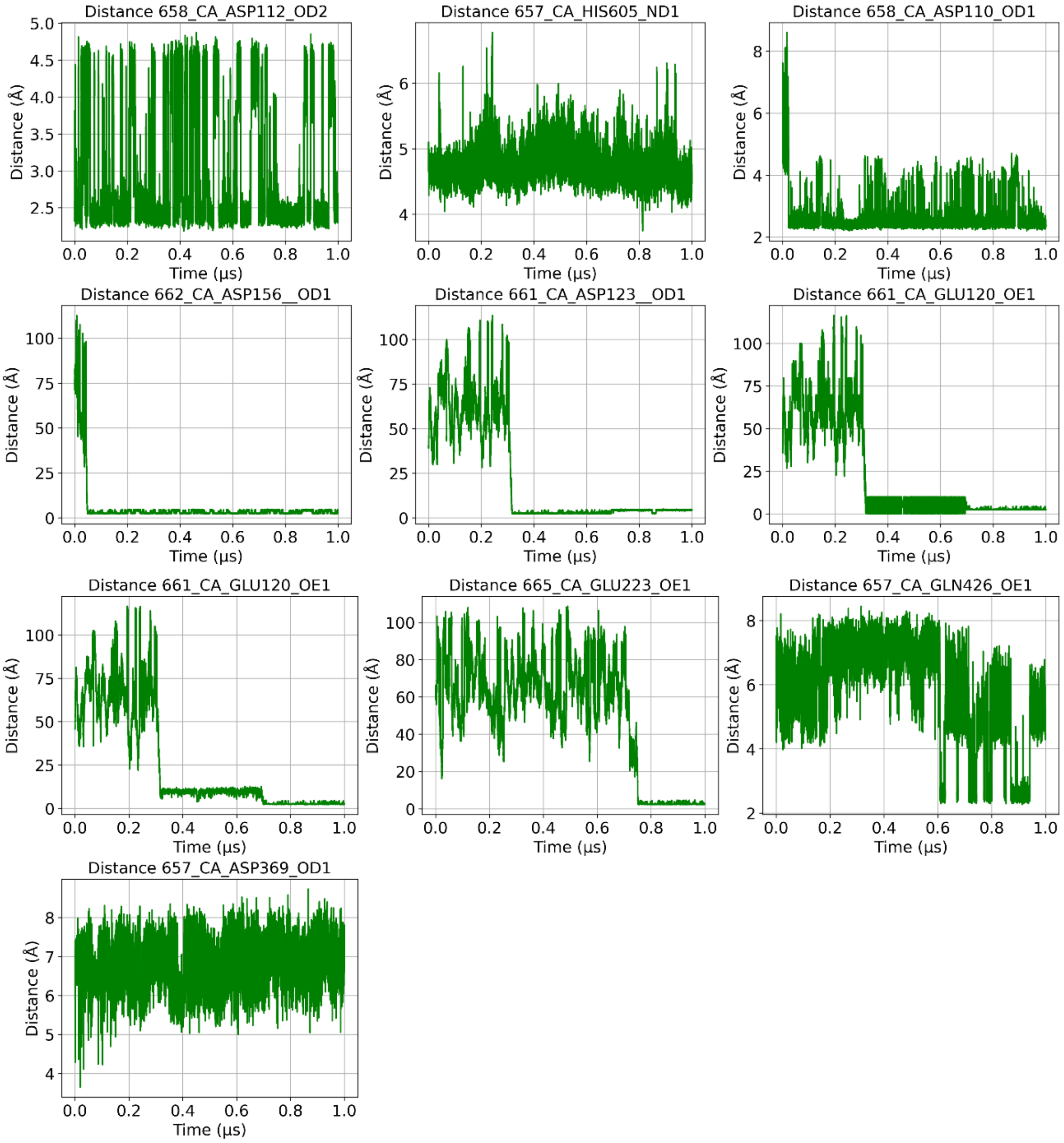

Figure S18. Distances between calcium and residues of the chimera enzyme at 0.01M Ca^{2+} concentration for replica 1.

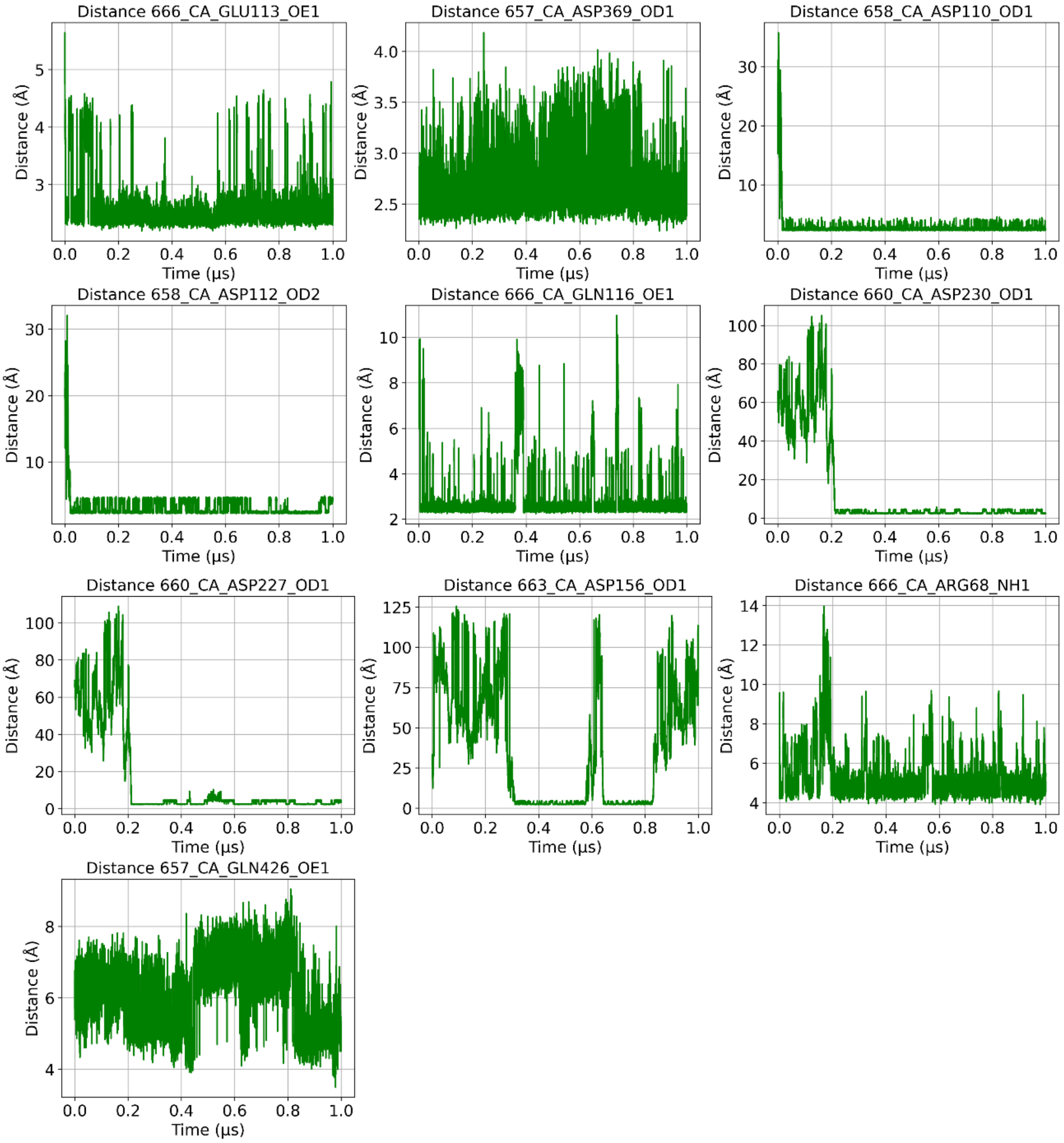

Figure S19. Distances between calcium and residues of the chimera enzyme at 0.01M Ca^{2+} concentration for replica 2.

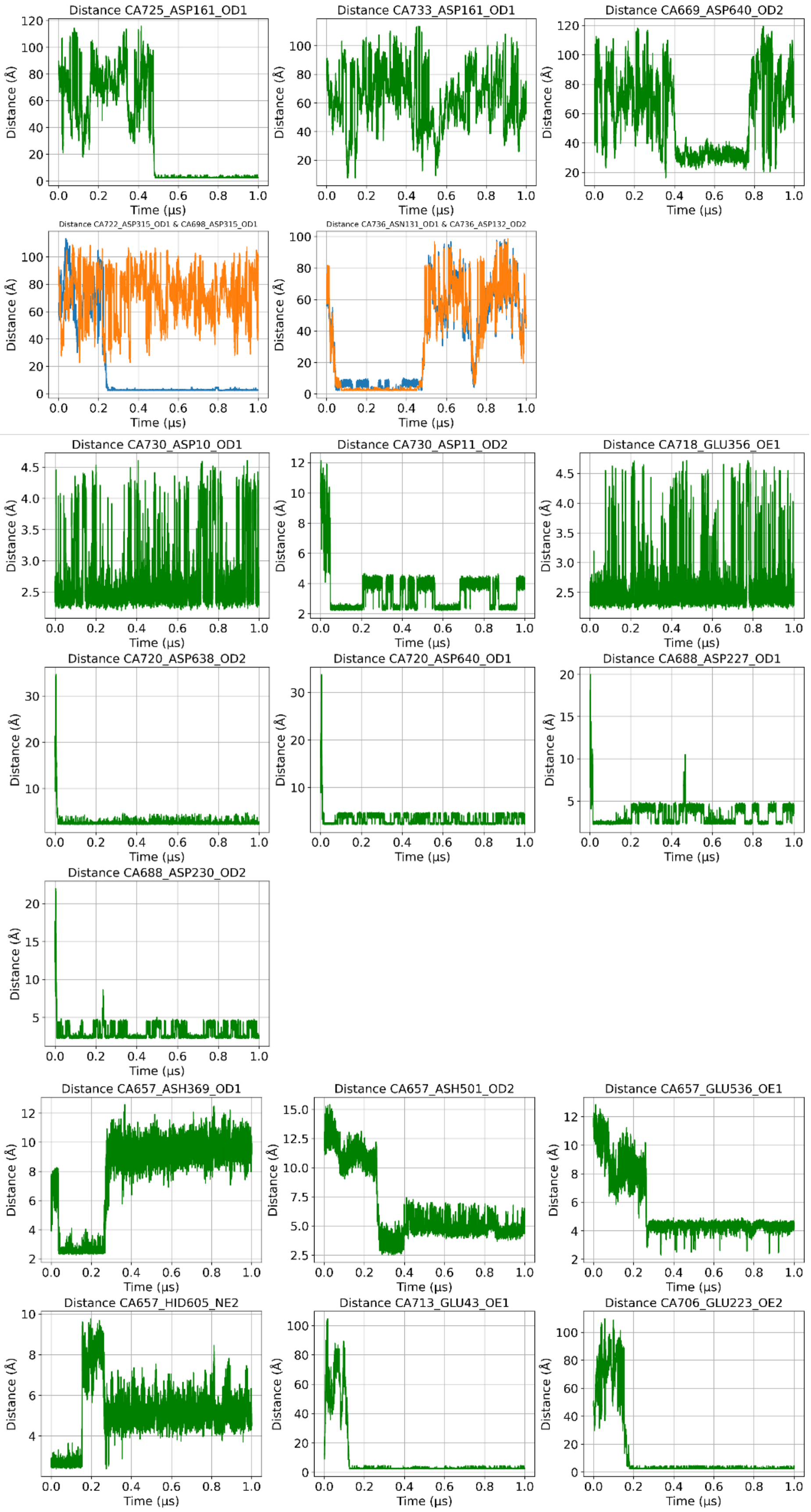

Figure S20. Distances between calcium and residues of the chimera enzyme at 0.1M Ca^{2+} concentration for replica 1.

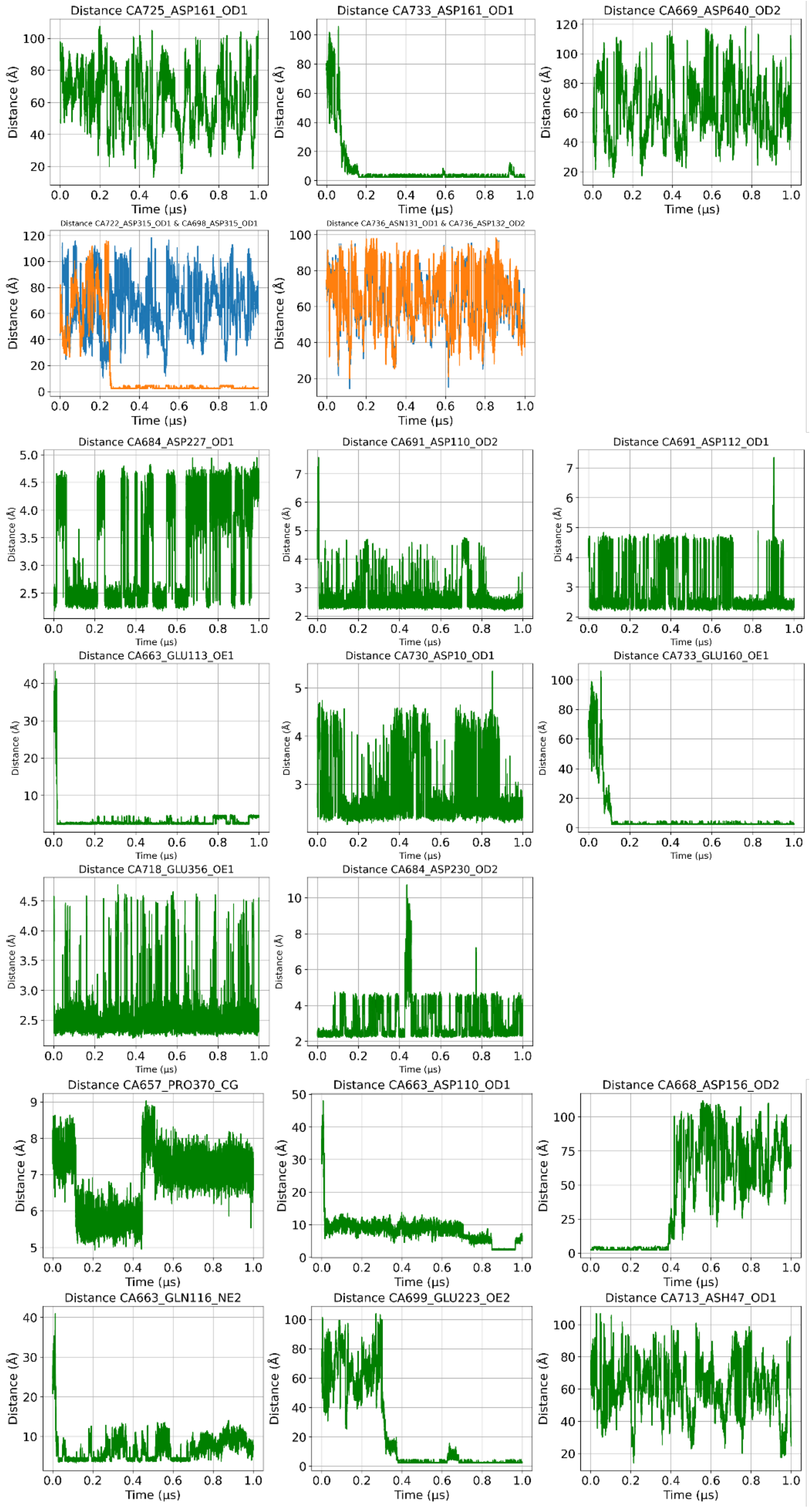

Figure S21. Distances between calcium and residues of the chimera enzyme at 0.1M Ca^{2+} concentration for replica 2.
